# RhoGDI1 regulates cell-cell junctions in polarized epithelial cells

**DOI:** 10.3389/fcell.2024.1279723

**Published:** 2024-07-17

**Authors:** Nicolina Wibbe, Tim Steinbacher, Frederik Tellkamp, Niklas Beckmann, Frauke Brinkmann, Manuel Stecher, Volker Gerke, Carien M. Niessen, Klaus Ebnet

**Affiliations:** ^1^ Institute-Associated Research Group “Cell Adhesion and Cell Polarity”, Institute of Medical Biochemistry, Zentrum für Molekularbiologie der Entzündung, University Münster, Münster, Germany; ^2^ Department Cell Biology of the Skin, University Hospital of Cologne, University of Cologne, Cologne, Germany; ^3^ Cologne Excellence Cluster on Stress Responses in Aging-associated Diseases (CECAD), University of Cologne, Cologne, Germany; ^4^ Institute of Medical Biochemistry, ZMBE, University Münster, Münster, Germany; ^5^ Cells-in-Motion Cluster of Excellence (EXC 1003—CiM), University of Münster, Münster, Germany; ^6^ Department Cell Biology of the Skin, University Hospital of Cologne, University of Cologne, Cologne, Germany; ^7^ Center for Molecular Medicine Cologne (CMMC), University Hospital of Cologne, University of Cologne, Cologne, Germany

**Keywords:** ARHGDIA, epithelial barrier, contact inhibition of locomotion, JAM-A, RhoGDI1, tight junction

## Abstract

Cell-cell contact formation of polarized epithelial cells is a multi-step process that involves the co-ordinated activities of Rho family small GTPases. Consistent with the central role of Rho GTPases, a number of Rho guanine nucleotide exchange factors (GEFs) and Rho GTPase-activating proteins (GAPs) have been identified at cell-cell junctions at various stages of junction maturation. As opposed to RhoGEFs and RhoGAPs, the role of Rho GDP dissociation inhibitors (GDIs) during cell-cell contact formation is poorly understood. Here, we have analyzed the role of RhoGDI1/ARHGDIA, a member of the RhoGDI family, during cell-cell contact formation of polarized epithelial cells. Depletion of RhoGDI1 delays the development of linear cell-cell junctions and the formation of barrier-forming tight junctions. In addition, RhoGDI1 depletion impairs the ability of cells to stop migration in response to cell collision and increases the migration velocity of collectively migrating cells. We also find that the cell adhesion receptor JAM-A promotes the recruitment of RhoGDI1 to cell-cell contacts. Our findings implicate RhoGDI1 in various processes involving the dynamic reorganization of cell-cell junctions.

## Introduction

Epithelia consist of sheets of cells with a pronounced apical-basal membrane polarity, which is reflected by three distinct membrane domains that differ in protein and lipid composition: an apical, non-bounded membrane domain that typically faces the lumen of an organ, a lateral membrane domains that adheres to adjacent cells, and a basal membrane domain that adheres to the extracellular matrix (Buckley and St Johnston, 2022). The development of membrane polarity requires the presence of an intramembrane diffusion barrier to prevent the intermixing of integral membrane components. In vertebrate epithelial cells, this diffusion barrier is localized at the most apical region of cell-cell contacts, the tight junctions (TJs) (Umeda et al., 2006).

The formation of TJs and the development of membrane polarity in response to cell-cell contact formation is a gradual process which is regulated by the concerted activities of cell adhesion receptors and cell polarity proteins (Macara, 2004; Ebnet, 2017). Initial contact sites, so-called puncta or primordial, spot-like adherens junctions (pAJs), are characterized by adhesion receptors like E-cadherin, JAM-A and nectin-2, and their associated scaffolding proteins including α-catenin, β-catenin, ZO-1 and afadin (Yonemura et al., 1995; Ando-Akatsuka et al., 1999; Ebnet et al., 2001; Suzuki et al., 2002). The subsequent localization of integral membrane proteins like occludin and claudin is followed by the recruitment of cell polarity proteins PAR-3, PAR-6, Lgl and aPKC (Suzuki et al., 2002; Yamanaka et al., 2003). The further maturation of immature, zipper-like junctions into mature, linear junctions with spatially separated TJs and AJs is mediated by aPKC (Suzuki et al., 2001; Suzuki et al., 2002) which triggers a reorganization of polarity complexes and their mutually exclusive localization through antagonistic interactions (Benton and St Johnston, 2003; Hurov et al., 2004; Suzuki et al., 2004). In fully polarized epithelial cells, an active PAR-6—aPKC complex localizes to the apical membrane domain, PAR-3 is localized at TJs, and the Lgl-containing Scribble complex as well as the polarity kinase PAR-1 localize to the lateral membrane domain (Buckley and St Johnston, 2022).

Many steps during junction formation and epithelial polarization are regulated by Rho family small GTPases (in short Rho GTPases). For example, in response to initial cell-cell contact formation Rac1 activity is downregulated at contacting areas to prevent continuous lamellipodial activity, a process called contact inhibition of locomotion (CIL) (Roycroft and Mayor, 2016). However, during maturation of pAJs, Cdc42 and/or Rac1 activities are required for the activation of aPKC at pAJs, which is necessary to trigger their maturation into linear junctions with separated TJs and AJs (Noda et al., 2001; Suzuki et al., 2001; Yamanaka et al., 2001). When cell polarization is complete, Cdc42 activity at the vertebrate marginal zone activates the PAR-6—aPKC complex to restrict PAR-3 to TJs (Zihni et al., 2014). In addition, Cdc42 at the apical membrane activates MRCK thereby stimulating apical myosin II activation and inhibiting lateral/junctional RhoA activity, which triggers an actomyosin contractility-based mechanism of PAR protein segregation (Zihni et al., 2017). Consistent with a role of Rho GTPases in epithelial polarization, several RhoGEFs and RhoGAPs have been identified at AJs and TJs (Citi et al., 2014; Mack and Georgiou, 2014; Braga, 2018; Ebnet and Gerke, 2022). The principal mechanism underlying the spatial regulation of the activities of Rho GTPases is their global delivery to membranes by GDP dissociation inhibitors (GDIs) and the regulation of their activities by locally resident RhoGEFs and RhoGAPs (Cherfils and Zeghouf, 2013).

As opposed to GEFs and GAPs the role of GDIs in regulating cell-cell contact formation and epithelial polarity is less well understood. Only three *RhoGDI* genes exist in mammals, which encode RhoGDI1/RhoGDIα (*ARHGDIA*), RhoGDI2/RhoGDIβ (*ARHGDIB*) and RhoGDI3/RhoGDIγ (*ARHGDIG*). RhoGDI1 is ubiquitously expressed (Xie et al., 2017) and interacts with several Rho GTPases including RhoA, RhoC, Rac1, Rac2, Rac3, RhoG and Cdc42 (Garcia-Mata et al., 2011; Tripathi et al., 2023). RhoGDI2 is mainly expressed in hematopoietic cells and in cancer cells and has a broad specificity for several Rho GTPases which is similar to RhoGDI1 (Griner and Theodorescu, 2012; Cho et al., 2019; Tripathi et al., 2023). RhoGDI3 is predominantly expressed in brain, testes and pancreas and shows a preferential activity towards RhoB and RhoG (de Leon-Bautista et al., 2016).

The main function of RhoGDIs is to extract the prenylated GDP-bound GTPases from membranes, either from the ER membrane for subsequent transport to their target membranes, or from the target membranes for subsequent sequestration in the cytoplasm and protection from proteasomal degradation (Garcia-Mata et al., 2011). At a given time, the vast majority of GTPases is maintained in an inactive form in the cytosol through GDIs (Garcia-Mata et al., 2011), implicating a rather passive role of RhoGDIs as a shuttle system to deliver and extract inactive Rho GTPases to and from membranes. Recent observations, however, show that RhoGDIs can extract active Rho GTPases to contribute to the spatial regulation of Cdc42 during cell wound repair (Golding et al., 2019). Also, in non-polarized cells RhoGDI1 has been found in association with various integral membrane proteins including Syndecan 4 (Elfenbein et al., 2009; Keller-Pinter et al., 2017), αvβ8 integrin (Reyes et al., 2013; Lee et al., 2015), EphrinB1 (Cho et al., 2018) and Plexin-B3 (Li and Lee, 2010), suggesting that RhoGDIs are localized at specific sites of cell-matrix and cell-cell adhesion to contribute to the spatial regulation of Rho GTPase activities.

In this study, we addressed the role of RhoGDI1 during cell-cell contact formation in polarized epithelial cells. We find that the depletion of RhoGDI1 deteriorates several processes associated with cell-cell junction formation including cell-cell contact maturation, development of the barrier function, collective cell migration, and CIL. We also find that the cell adhesion receptor JAM-A promotes the recruitment of RhoGDI1 to cell-cell junctions. Our observations suggest that the function of RhoGDI1 activity is required during several processes that involve the dynamic reorganization of cell-cell junctions.

## Results

### Depletion of RhoGDI1 delays cell-cell contact formation in polarized epithelial cells

**FIGURE 1:**
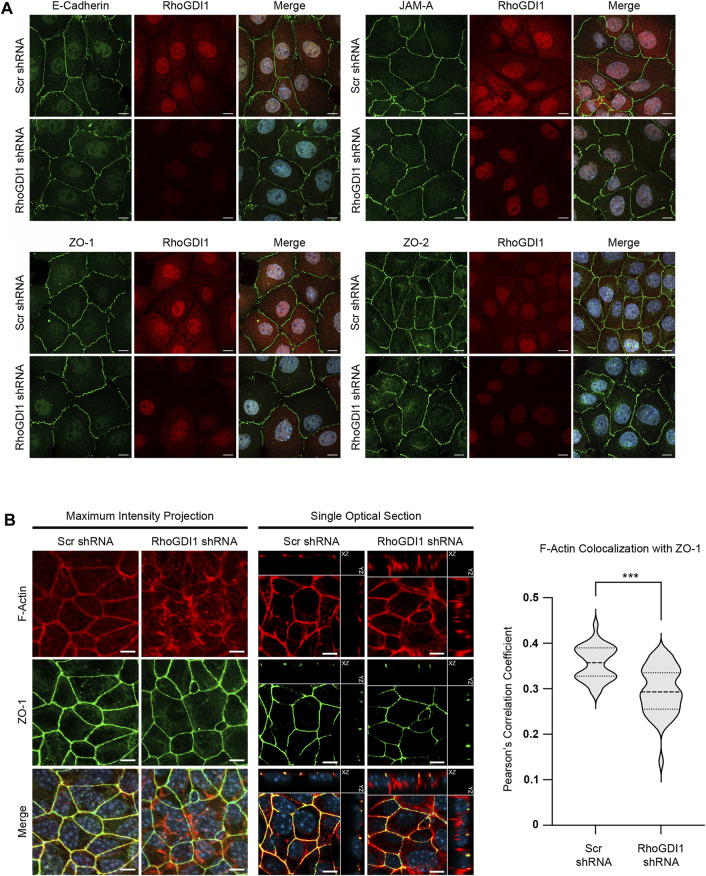
RhoGDI1 depletion does not alter the localization of proteins associated with AJs and TJs but alters F-actin distribution. **(A)** Immunofluorescence analysis of cell-cell junctions of RhoGDI1-depleted Eph4 cells. Eph4 cells expressing scrambled shRNAs (Ctrl KD) and Eph4 cells expressing RhoGDI1 shRNAs (RhoGDI1 KD) were fixed and stained with antibodies against RhoGDI1 together with antibodies against E-cadherin, JAM-A, ZO-1 or ZO-2 as indicated. Note that all AJ- and TJ-localized proteins are normally localized at cell-cell contacts of RhoGDI1 KD Eph4 cells. Scale bars: 10 µm. **(B)** Immunofluorescence analysis of Eph4 cells transfected with scrambled shRNA (Scr shRNA) or RhoGDI1-specific shRNAs (RhoGDI1 shRNA) were grown on polycarbonate filters to enhance apico-basal polarization and stained with antibodies against ZO-1 and with fluorescently labeled phalloidin to visualize F-actin as indicated. Maximum intensity projections (scale bars: 5 µm) and single optical sections (scale bars: 5 µm) are shown. In single optical sections, XZ and YZ projections are separated from the XY projections by solid lines. Right panel: Quantification of F-actin colocalization with ZO-1. The F-actin and ZO-1 fluorescence intensities were analyzed using Imaris software. Data shows the colocalization of F-actin fluorescence with the ZO-1 fluorescence and is depicted as Pearson’s correlation coefficient. Statistical analysis was performed with unpaired, two-tailed Student’s t-test. Data was obtained and data points were pooled from at least 10 randomly chosen fields of view (FOV) per experiment derived from three independent experiments (Scr shRNA: 12, 12, 11 FOVs; RhoGDI1 shRNA: 10, 15, 15 FOVs), with each FOV containing approximately 100 cells. Data is presented as Violin plot. Bold broken lines indicate median values, thin broken lines indicate first and third quartiles. ***p < 0.001.

Based on the important function of Rho family small GTPases in junction formation and maturation in polarized epithelial cells (Braga, 2018; Ebnet and Gerke, 2022), we analyzed the role of RhoGDI1 in Eph4 cells, a murine mammary gland-derived cell line that develops apical-basal polarity with well-developed tight junctions (TJs) (Umeda et al., 2006). We stably expressed RhoGDI1-specific shRNAs in Eph4 cells (Supplementary Figure S1) and analyzed the localization of several integral membrane and peripheral membrane proteins localized at AJ and TJ, including E-cadherin, JAM-A, ZO-1 and ZO-2. The localization of these proteins was unchanged after depletion of RhoGDI1 (Figure 1A) suggesting that cell-cell junctions can form in the absence of RhoGDI1. F-actin, which was concentrated at cell-cell contact regions in Eph4 WT cells, was diffusely localized in the cytoplasm in RhoGDI1-depleted cells (Figure 1B), which is in line with previous studies showing altered F-actin patterns in various cell types after depletion of RhoGDI1 (Rivero et al., 2002; Chinchole et al., 2022; Zhu et al., 2023). Consistent with the role of RhoGDI1 as a chaperone for Rho GTPases (Garcia-Mata et al., 2011), and similar to what has been observed previously in other cell types (Gorovoy et al., 2007; Boulter et al., 2010; Gupta et al., 2013; Chinchole et al., 2022), the protein levels of Cdc42, Rac1 and RhoA were reduced after depletion of RhoGDI1 (Supplementary Figure S2). Together, these findings suggested that RhoGDI1 depletion in Eph4 cells results in alterations in F-actin localization and reduced protein levels of Rho family GTPases without affecting the ability of the cells to develop mature cell-cell junctions.

Recent observations indicated that RhoGDI1 is downregulated by TGFβ1, which is a major inducer of epithelial-to-mesenchymal transition (EMT) (Huang et al., 2021). To test if RhoGDI1 depletion induces a mesenchymal phenotype in Eph4 cells we analyzed the levels of the intermediate filament protein vimentin and of the transcription factor Snail2/Slug, two proteins known to be upregulated during EMT (Lamouille et al., 2014). We observed no difference in the expression levels of these two EMT marker proteins after depletion of RhoGDI1 (Supplementary Figure S3), suggesting that the loss of RhoGDI1 expression does not induce alterations associated with EMT in Eph4 cells.

**FIGURE 2:**
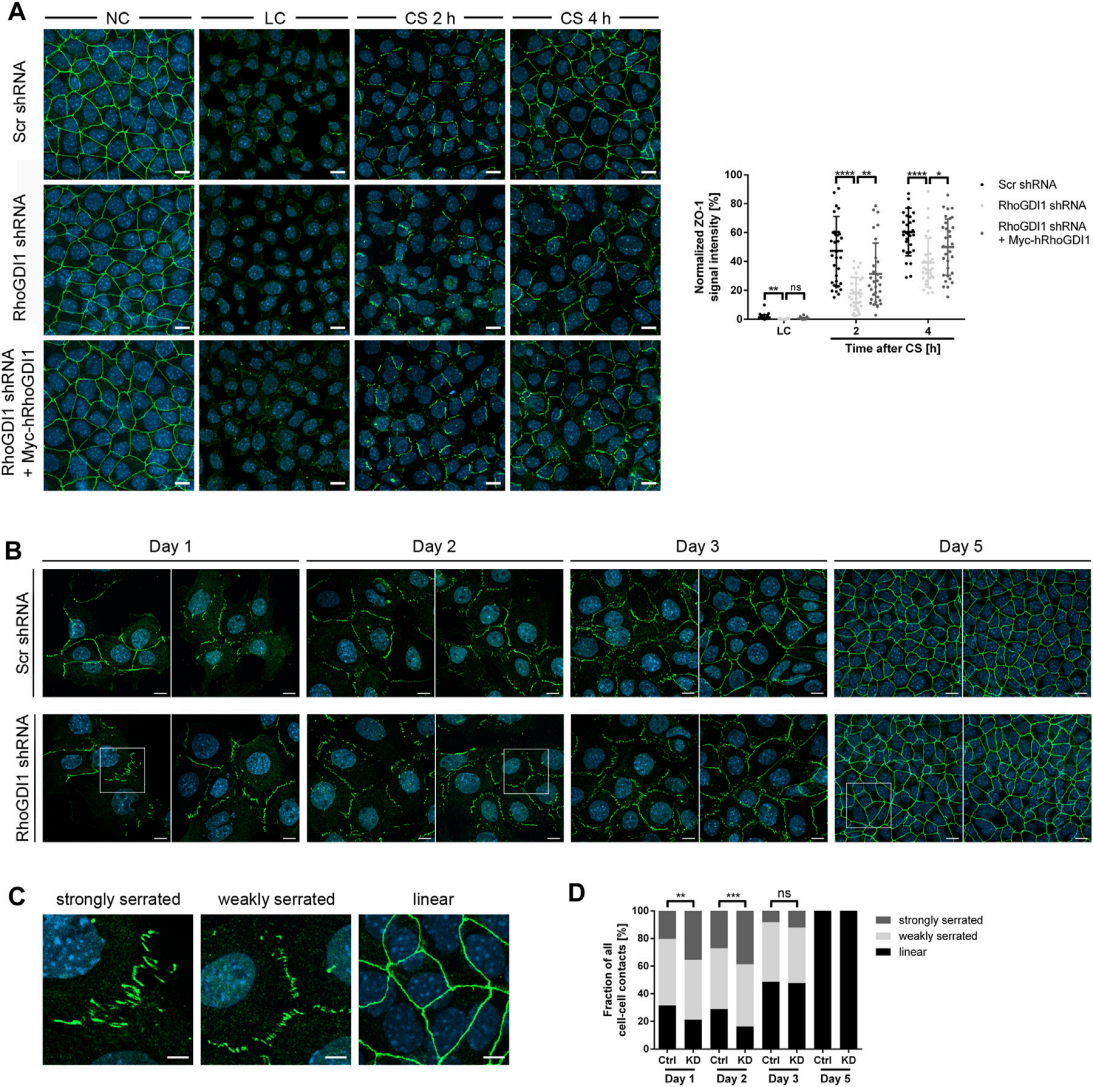
RhoGDI1 is required for the timely maturation of cell-cell contacts. **(A)** Eph4 cells (Scr shRNA, RhoGDI1 shRNA, RhoGDI1 shRNA + Myc-hRhoGDI1) were either cultured under normal Ca^2+^ conditions (NC) or were cultured under low Ca^2+^ conditions (LC) and either fixed immediately (LC) or supplemented with normal medium (Ca^2+^ switch, CS) for 2 h or 4 h. Cells were stained for ZO-1 (green fluorescence) as marker for cell-cell contacts (Yonemura et al., 1995) and for DNA (DAPI, blue fluorescence). Left panels: Representative ZO-1 immunofluorescence pictures taken at the indicated time point before and after CS. Scale bars: 10 µm. Right panel: Quantification of ZO-1 localization at different time points after CS as indicated. The ZO-1 IF intensities were analyzed using ImageJ software, data were normalized to the ZO-1 intensities observed in cells grown under normal Ca^2+^ conditions (NC, 100%). Statistical analysis was performed with unpaired, two-tailed Student’s t-test. Data is derived from at least 30 independent fields of view derived from three independent experiments. Data are presented as mean values ± SD. ns, not significant; *p < 0.05, **p < 0.01, ****p < 0.0001. Abbreviations: NC, normal Ca^2+^ conditions; LC, low Ca^2+^ conditions; CS, Ca^2+^ switch. **(B)** Eph4 cells (Scr shRNA, RhoGDI1 shRNA) seeded at low density were grown for different time periods, then fixed and stained for ZO-1 (green fluorescence) to visualize cell-cell junctions and with DAPI (blue fluorescence) to visualize nuclei. Two representative images are shown for each condition. Squares indicate areas shown at higher magnifications in **(C)**. Scale bars: 10 µm. **(C)** Representative images of junction phenotypes arbitrarily defined as strongly serrated, weakly serrated, and linear. Scale bars: 5 µm. **(D)** Quantification of junction phenotypes. Classification of junction phenotypes was performed as detailed in the Methods section. Statistical analysis was performed with Chi-Square test. Data is derived from at least 10 randomly chosen fields of view in each experiment with a total number of at least 187 cell-cell contacts analyzed. Data is derived from three independent experiments. ns, not significant, **p < 0.01, ***p < 0.001.

The formation of cell-cell junctions requires the concerted activities of several Rho family GTPases (Braga, 2018). To analyze the role of RhoGDI1 during cell-cell contact formation confluent monolayers of RhoGDI-depleted Eph4 cells and of RhoGDI-depleted Eph4 cells expressing Myc-hRhoGDI1 were subjected to a Ca^2+^-switch (CS) to induce new cell-cell contact formation (see Method section for details). Cells were fixed at different time points after CS and analyzed for the localization of ZO-1, a peripheral membrane protein which is present at the earliest sites of cell-cell contacts and which remains junction-associated during cell polarization (Yonemura et al., 1995). Depletion of RhoGDI1 resulted in reduced ZO-1 fluorescent signals at cell-cell contacts both 2 h and 4 h after CS (Figure 2A). Ectopic expression of Myc-hRhoGDI1 significantly restored junctional ZO-1 fluorescence intensity. The fluorescent signals for JAM-A, a cell-cell adhesion receptor which is localized at primordial, spot-like adherens junctions (also called punctate adherens junctions, pAJs) during early steps of cell contact formation and which is stably associated with mature cell-cell junctions in polarized epithelial cells (Suzuki et al., 2002; Iden et al., 2012), were similarly reduced after depletion of RhoGDI1 and restored in cells expressing Myc-hRhoGDI1 (Supplementary Figure S4). These observations indicated that the activity of RhoGDI1 is required for the timely maturation of intercellular junctions in polarized epithelial cells.

During cell-cell contact formation, intercellular junctions gradually mature from pAJs via perpendicularly-oriented adherens junctions (serrated or discontinuous AJs) to thick linear junctions (Yonemura et al., 1995). Serrated junctions have been associated with active or remodeling junctions both in polarized epithelial cells and in endothelial cells (Taguchi et al., 2011; Bentley et al., 2014). To analyze the role of RhoGDI1 in cell-cell contact formation in more detail, we analyzed junction maturation over several days. Using ZO-1 as a marker for junction formation, intercellular junctions were classified as weakly serrated, strongly serrated, and linear (see Method section for details). Depletion of RhoGDI1 resulted in a significant increase in strongly serrated junctions at days 1 and 2 after seeding (Figures 2B–D). At day 3, the frequency of strongly serrated junctions was reduced, and at day 5 all cell-cell junctions had matured into linear cell-cell contacts, irrespective of the depletion of RhoGDI1 (Figures 2B–D). These observations indicated that RhoGDI1 is required for the timely development of serrated junctions into linear junctions, which is consistent with the necessity to downregulate Rho GTPase activity to reduce junction dynamics in order to enable the development of linear junctions (Citi et al., 2014; Roycroft and Mayor, 2016).

### RhoGDI1 regulates the barrier function of polarized epithelial cells

**FIGURE 3:**
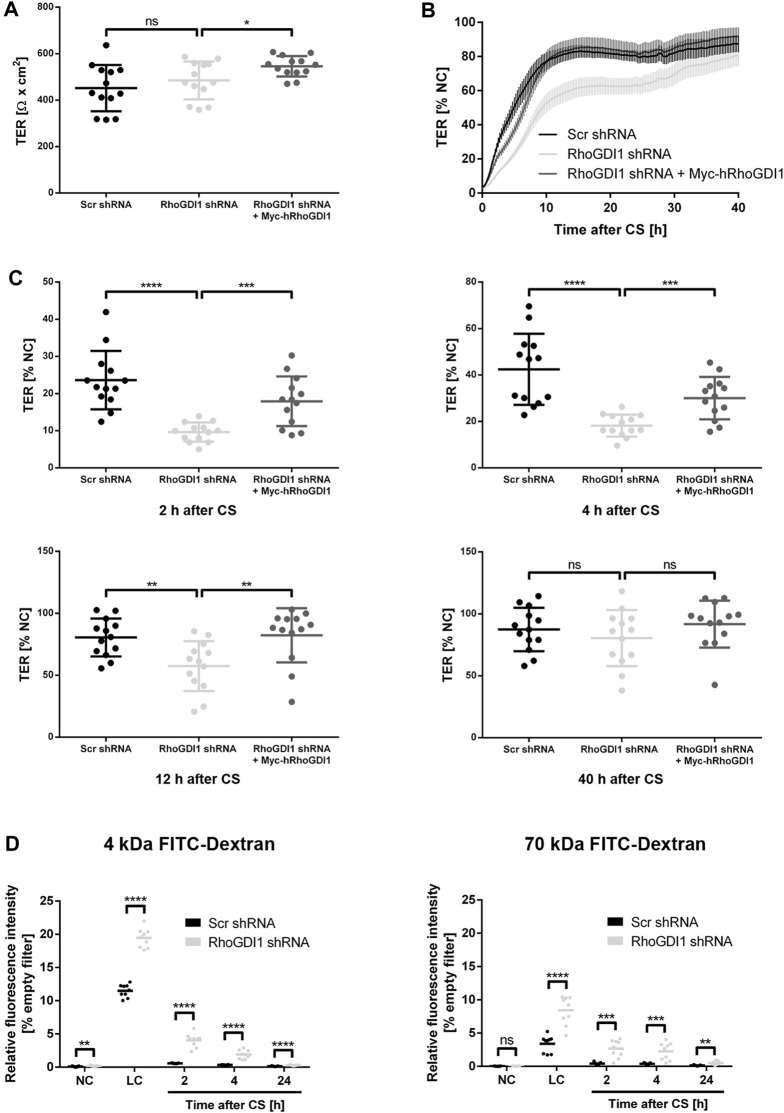
RhoGDI1 is required for the timely development of the epithelial barrier. **(A–C)** TER analysis of Eph4 cells expressing either a scrambled shRNA (Scr shRNA), a RhoGDI1 shRNA (RhoGDI1 shRNA) or a RhoGDI1 shRNA together with a shRNA-insensitive human RhoGDI1 construct (RhoGDI1 shRNA + Myc-hRhoGDI1). Cells were grown on polycarbonate filters. TER was measured using an automated multi-well device as described in the Methods section. **(A)** TER at steady state. Data are presented as mean values ± SD. **(B)** TER after CS. Cells were grown to confluency on polycarbonate filters and subjected to CS to induce new cell-cell contact formation. TER was recorded over 40 h after CS using an automated multi-well device and is depicted as percentage of the TER measured under normal Ca^2+^ conditions (NC). Data are presented as mean values ± SEM. **(C)** Quantification of TER values measured 2 h, 4 h, 12 h, and 40 h after CS. Data are presented as mean values ± SD. **(D)** Paracellular permeability for FITC dextran. Cells grown to confluency on polycarbonate filters were subjected to CS to induce new cell-cell contact formation as described above. The concentration of FITC-labelled dextran (4 kDa FITC-dextran, 70 kDa FITC-dextran) in the lower compartments was measured at the indicated time points. Data is indicated as the FITC fluorescence relative to the FITC fluorescence measured in samples with empty filters. Data are presented as mean values. Statistical analyses in this figure were performed with unpaired, two-tailed Student’s t-test. Data is derived from four independent experiments **(A–C)** or three **(D)** independent experiments using at least triplicate measurements in each experiment. Individual filters are represented by individual symbols in the graphs. ns, not significant, *p < 0.05, **p < 0.01, ***p < 0.001, ****p < 0.0001. Abbreviations: NC, normal Ca^2+^ conditions; LC, low Ca^2+^ conditions, CS, Ca^2+^ switch.

Cell-cell contact formation of polarized epithelial cells is a step-wise process during which pAJs mature to form functional cell-cell junctions with TJs separated from AJs (Tsukita et al., 2001). The maturation of pAJs requires the activation of aPKC by Rac1 and/or Cdc42 (Suzuki et al., 2002; Suzuki and Ohno, 2006). Thus, despite the necessity to downregulate Rac1 activity in response to initial contact formation to prevent continuing protrusive activity (Roycroft and Mayor, 2016), the activity of Rho family small GTPases is necessary to promote junctional maturation and to establish barrier-forming tight junctions (Braga, 2018; Ebnet and Gerke, 2022). To address the role of RhoGDI1 during this process, we analyzed the development of transepithelial electrical resistance (TER) as a read-out for the barrier function using impedance spectroscopy (Wegener et al., 2004). Under steady state conditions, we observed no TER difference between control cells and RhoGDI1 KD cells (Figure 3A). When cells were subjected to a CS to induce new contact formation, RhoGDI1-depleted cells showed a significant delay in the establishment of TER, which was restored upon expression of Myc-hRhoGDI1 (Figures 3B,C). To verify a defect in barrier formation after depletion of RhoGDI1, we measured the permeability of RhoGDI1-depleted Eph4 cells for fluorescein isothiocyanate (FITC)-labeled tracer molecules of different molecular weights. Depletion of RhoGDI1 resulted in a strong increase in the permeability for 4 kDa and 70 kDa FITC-dextran when cells were analyzed 2 h and 4 h after CS (Figure 3D). 24 h after CS cells had established a normal barrier for both FITC-dextran tracer molecules. Together these findings indicated that the activity of RhoGDI1 is required for the establishment of barrier-forming TJs. They also suggest that despite the necessity of active Rho GTPases for junction maturation (Braga, 2018), their activity must be tightly regulated to allow a timely formation of the epithelial barrier.

### RhoGDI1 is required to establish contact inhibition of locomotion in colliding cells

**FIGURE 4:**
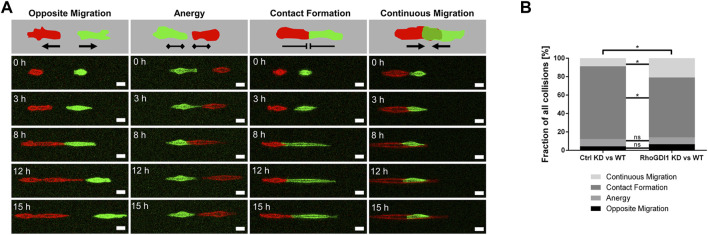
RhoGDI1 regulates contact inhibition of locomotion. **(A)** 1D kinematic CIL assay. Ctrl Eph4 cells or RhoGDI1 KD Eph4 cells (labeled with LA-EGFP) were co-cultured with WT Eph4 cells (labeled with LA-mCherry) on linear micropatterns (width: 5 µm) and observed by live microscopy for 15 h. Cartoons: Types of CIL behavior after cell-cell collision: Opposite migration, Anergy, Contact Formation, Continuous Migration. Bottom panels: Still images of movies representative for different types of CIL behavior. Scale bars: 20 µm. **(B)** Quantification of CIL types after cell collisions of scrambled shRNA-expressing Eph4 cells with WT Eph4 cells (Ctrl KD vs. WT) and of RhoGDI1 shRNA-expressing Eph4 cells with WT Eph4 cells (RhoGDI1 KD vs. WT). Statistical analysis was performed with Chi-Square test with Bonferroni corrections. Data are presented as mean values. ns, not significant, *p < 0.05. Number of collisions: n = 138 for Ctrl KD–WT (4 independent experiments), n = 163 for RhoGDI1 KD–WT (4 independent experiments).

The delay in junction formation and maturation after RhoGDI1 depletion suggested that RhoGDI1-depleted cells fail to balance the activity of Rho GTPases during cell-cell contact-dependent processes. When migrating epithelial cells encounter other cells of the same type, as it occurs during mesenchymal-epithelial transitions or during wound healing, they downregulate Rac1 activity at their leading edge to prevent continuing protrusion formation and migration, a process called contact inhibition of locomotion (CIL) (Roycroft and Mayor, 2016). A failure in regulating CIL results in the migration of cells across collided cells and has been implicated in cancer invasion (Stramer and Mayor, 2017). We have previously observed that depletion of the cell adhesion receptor JAM-A in tumor cells results in a loss of CIL associated with high Rac1 activity at sites of cell-cell contacts between colliding cells (Kummer et al., 2022). To analyze the role of RhoGDI1 during CIL, we used a micropattern-based collision assay in which single cells are grown on functionalized stripes of defined widths (1D kinematic assay) (Scarpa et al., 2013; Schwietzer et al., 2023). Compared with regular (2D) culture conditions, this approach limits the degree of freedom of motion thus forcing cell-cell interactions, which facilitates interpretation (Stramer and Mayor, 2017). Co-cultures of either control Eph4 cells or RhoGDI1 KD Eph4 cells (labeled with LA-GFP) with Eph4 WT cells (labeled with LA-mCherry) were grown on micropatterned stripes of 5 µm width and observed by live cell microscopy over a period of 15 h. Post-collision events were categorized as “opposite migration” (Type −2), “anergy” (Type −1), “contact formation” (Type 0), and “continuous migration” (Type +1). RhoGDI1-depleted cells less frequently formed stable cell-cell contacts and instead more frequently migrated across the collided cells (Figures 4A,B). These findings indicate the RhoGDI1 is required to prevent continuing migration in response to cell-cell collision, which is a central aspect of CIL (Stramer and Mayor, 2017).

### RhoGDI1 regulates collective cell migration of polarized epithelial cells

**FIGURE 5:**
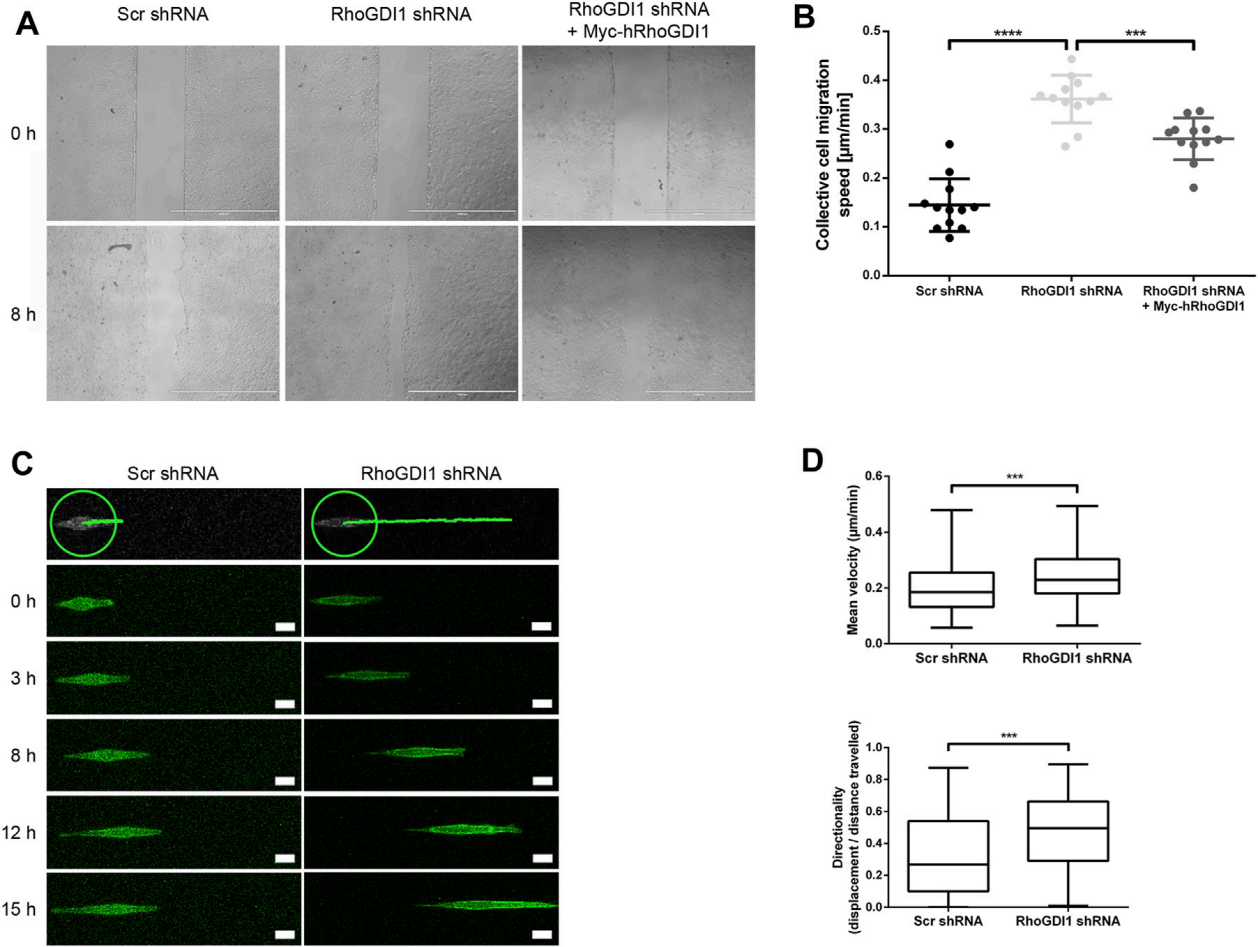
RhoGDI1 regulates cell migration of Eph4 cells. **(A)** Monolayer expansion assays of scrambled shRNA-transfected Eph4 cells (Scr shRNA), RhoGDI1 shRNA-transfected Eph4 cells (RhoGDI1 shRNA), and RhoGDI1 shRNA-transfected Eph4 cells expressing a shRNA-insensitive hRhoGDI1 construct (RhoGDI1 shRNA + Myc-hRhoGDI1). Cells were seeded on FN-coated microscope slides separated by a silicone stamp. Collective cell migration was triggered by stamp removal. The migration velocity of the cell collective was quantified by measuring the cell-free area directly after the removal of the stamp and 8 h later (see Methods section for details). Representative images of monolayer expansion immediately after stamp removal (0 h) and after 8 h (8 h). Scale bars: 1000 µm. **(B)** Quantifications of collective cell migration velocities. Statistical analysis was performed with unpaired, two-tailed Student’s t-test. Each dot represents one biological replicate (one independent cell population). Data are presented as mean values ± SD. Number of independent cell populations analyzed: n = 12 for each cell line (3 independent experiments). ***p < 0.001, ****p < 0.0001. **(C)** 1D kinematic assays of RhoGDI1-depleted cells. Control cells (Scr shRNA) and RhoGDI1-depleted cells (RhoGDI1 shRNA) expressing LA-EGFP were cultured on linear micropatterns (width: 5 µm) and observed by live microscopy for 15 h. Still images show single cells migrated on linear tracks at different time points.The top panels show the distances covered by the cells after the observation period. The green circles indicate the positions of the cells at the beginning of the observation period. Scale bars: 20 µm. **(D)** Quantification of mean velocity (top panel) and directionality (bottom panel) of single cells cultured on linear micropatterns. Analysis was performed using the TrackMate Plugin for ImageJ software. Statistical analysis was performed with two-sided Mann-Whitney *U*-Test. Number of cells analyzed: n = 101 (Scr shRNA) and n = 91 (RhoGDI1 shRNA) (3 independent experiments). ***p < 0.001.

Polarized epithelial cells can migrate as sheets in which individual cells are connected through intercellular adhesive interactions (Friedl and Mayor, 2017). During migration, forces generated both by cell-matrix adhesion and cell-cell adhesion are sensed by cell-cell adhesion receptors, which transmit these forces to their neighbors through their association with the underlying actomyosin cytoskeleton (Mayor and Etienne-Manneville, 2016). In addition, cells generate polarized protrusions beneath the cells migrating in front of the cell, so-called cryptic lamellipodia, which requires the Arp2/3 complex and its regulator Wave localized at adherens junctions (Farooqui and Fenteany, 2005; Ozawa et al., 2020). Given the important role of Rho GTPases in regulating actomyosin contractility and lamellipodia formation during collective cell migration, we addressed the role of RhoGDI1 in collectively migrating Eph4 cells. To this end we used a monolayer expansion model in which cells are grown in two-chamber slides where individual chambers are separated by a removable insert (Das et al., 2015; Tholmann et al., 2022). Following removal of the confinement which separates the two chambers, migration of the monolayer was monitored over a period of 8 h RhoGDI1-depleted Eph4 cells migrated significantly faster than control cells (Figures 5A,B), and ectopic expression of shRNA-insensitive human RhoGDI1 construct partially reversed the increased migration velocities. These observations indicated that RhoGDI1 contributes to the regulation of migration when polarized epithelial cells migrate as sheets, which is consistent with a role of RhoGDI1 in limiting the activities Rho GTPases.

To address the question if the increased migratory speed of collectively migrating cells after RhoGDI1 depletion depends on RhoGDI1’s role in regulating cell-cell contact-dependent processes (Farooqui and Fenteany, 2005; Ozawa et al., 2020), we analyzed the migration of single cells grown on micropatterns. We found that both the migration velocity and the directionality of migration were increased in RhoGDI1-depleted cells (Figures 5C,D). These observations suggest a cell-autonomous role of RhoGDI1 in limiting cell motility that is independent of cell-cell contacts.

### The cell adhesion receptor JAM-A promotes the recruitment of RhoGDI1 to cell-cell contacts

**FIGURE 6:**
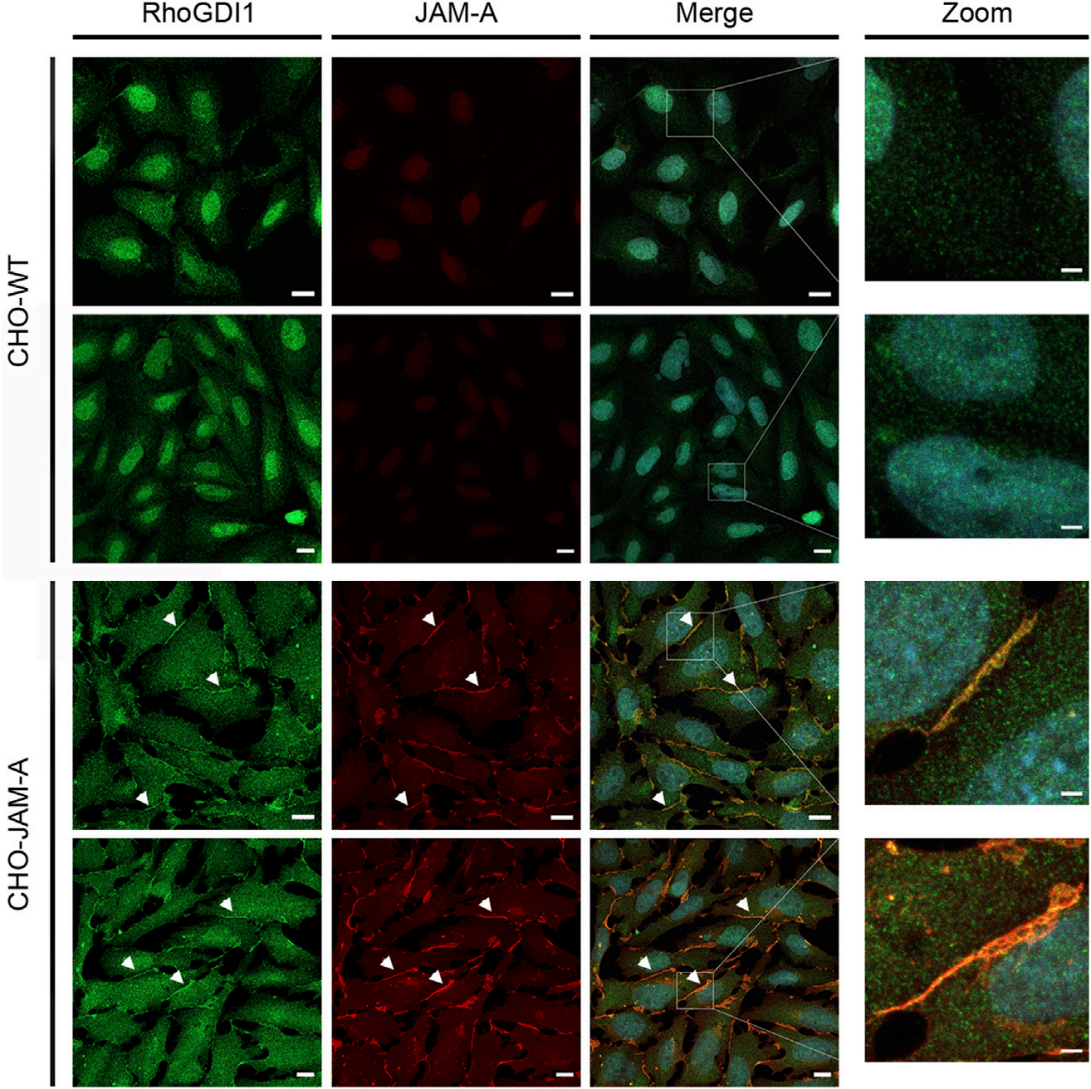
RhoGDI1 is recruited to JAM-A-based cell-cell contacts in CHO cells. (**A**) CHO WT cells (CHO-WT) or CHO cells stably expressing mJAM-A (CHO-JAM-A) were stained with antibodies against RhoGDI1 and JAM-A as indicated. Arrowheads indicate RhoGDI1-poitive cell-cell contacts. Right panels show magnifications of the insets marked by white squares in the merged images. Data is representative for four independent experiments. Scale bars: 10 µm (regular images), 2 µm (zoomed insets).

Given the dynamic regulation of Rho family small GTPases at cell-cell contacts during junction formation and during the development of membrane polarity we addressed the question if cell adhesion receptors of the JAM family recruit RhoGDI1 to cell-cell contacts. We used CHO cells since these cells lack classical adhesion receptors including JAMs, cadherins or nectins. Ectopic expression of these cell adhesion receptors results in the recruitment of cytoplasmic binding partners to cell-cell junctions (Ebnet et al., 2001; Ebnet et al., 2003; Miranda et al., 2003; Iden et al., 2006; Meng et al., 2019), making these cells a suitable system to analyze the ability of a single adhesion receptor to recruit cytoplasmic proteins to cell-cell contacts. In non-transfected CHO cells RhoGDI1 was not detectable at cell-cell contacts. In CHO cells expressing JAM-A, however, RhoGDI1 strongly co-localized with JAM-A at cell-cell contacts (Figure 6) indicating that JAM-A can recruit RhoGDI1 to cell-cell junctions.

**FIGURE 7:**
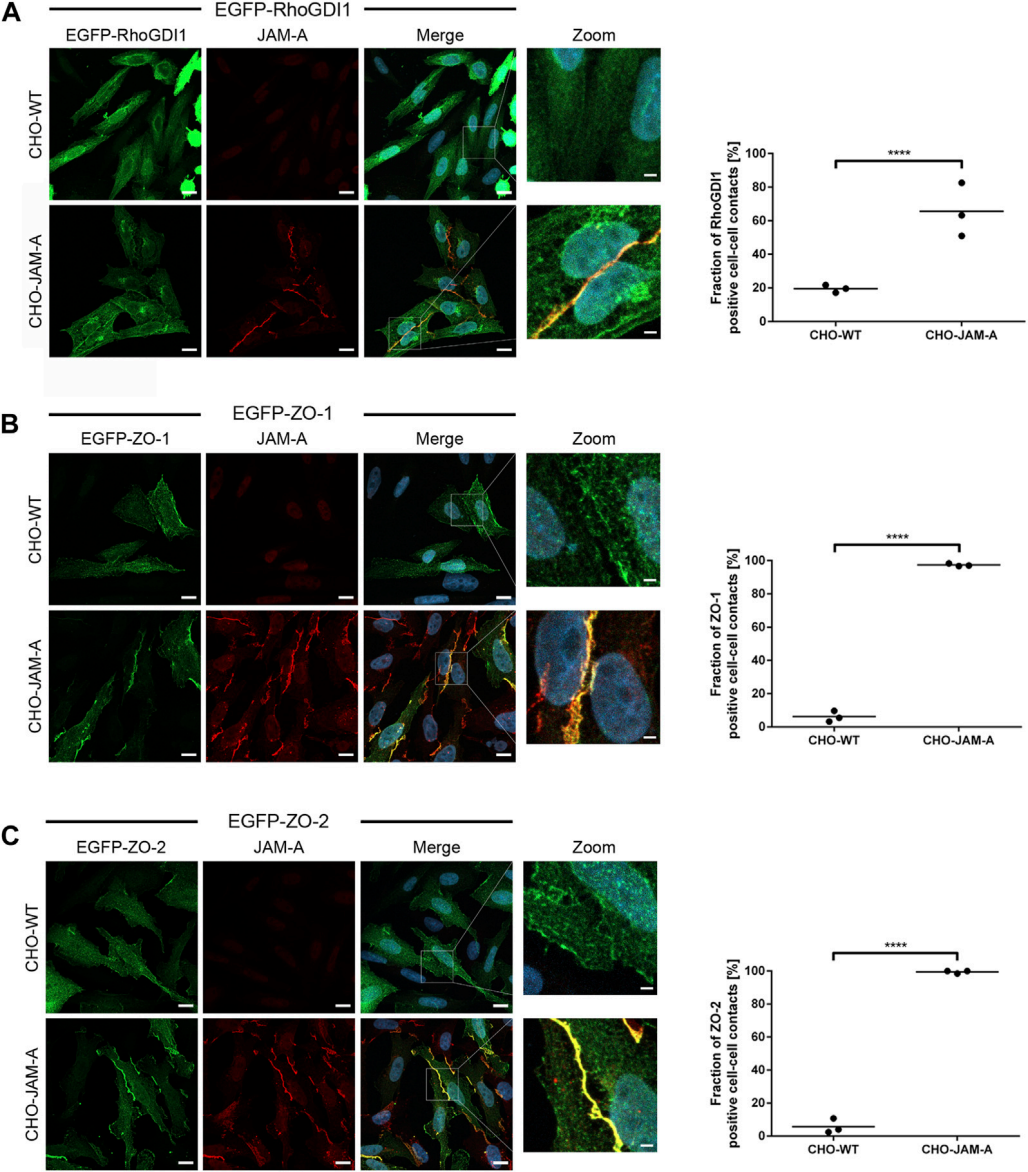
Quantification of RhoGDI1 recruitment by JAM-A. CHO WT cells (CHO-WT) or CHO cells stably expressing mJAM-A (CHO-JAM-A) were transiently transfected with either EGFP-RhoGDI1 **(A)**, EGFP-ZO-1 **(B)** or EGFP-ZO-2 **(C)**. Cells were stained with antibodies against JAM-A and with DAPI. Zoom pictures show high magnifications of the areas depicted by white squares in the merged images. Scale bars: 10 µm (regular images), 2 µm (insets). Plots show quantifications of cell-cell contact localization of EGFP-RhoGDI1, EGFP-ZO-1 and EGFP-ZO-2. In control cells (CHO-WT), cell-cell contact sites were identified on the basis of the cytoplasmic EGFP signal. Only cell-cell contact sites which showed no intercellular gaps on the basis of the EGFP fluorescence signals were included (examples are shown in the high magnification (Zoom) images. In JAM-A-transfected cells (CHO-JAM-A), cell-cell contact sites were identified on the basis of the junctional JAM-A fluorescence signals. Graphs show the fraction of EGFP-positive cell-cell contacts. Statistical analysis was performed with Fisher’s test. Data is derived from N = three independent experiments, each data point represents the mean value of one experiment. Number of analyzed cell-cell contacts: **(A)** EGFP-RhoGDI1: n (CHO-WT) = 131, n (CHO-JAM-A) = 155; **(B)** EGFP-ZO-1: n (CHO-WT) = 115, n (CHO-JAM-A) = 192; **(C)** EGFP-ZO-2: n (CHO-WT) = 128, n (CHO-JAM-A) = 162. ****p < 0.0001.

To further confirm the recruitment of RhoGDI1 by JAM-A, we transfected JAM-A-expressing CHO cells with EGFP-RhoGDI1 and analyzed its recruitment to JAM-A-based cell-cell contacts in comparison with ZO-1 and ZO-2, two scaffolding proteins which interact with JAM-A (Bazzoni et al., 2000; Ebnet et al., 2000; Monteiro et al., 2013). In WT CHO cells, EGFP-RhoGDI1 was detected as a faint signal at cell-cell contacts in approx. 20% of cell junctions (Figure 7A) and was not detectable in the vast majority of cells. In JAM-A CHO cells, EGFP-RhoGDI1 showed a strong signal in more than 60% of cell-cell contacts (Figure 7A). EGFP-ZO-1 and EGFP-ZO-2 were hardly detectable at cell-cell contacts of WT CHO cells (less than 5% of cell-cell contacts, very faint signals) but were detectable as strong signals in more than 95% of cell-cell contacts in JAM-A CHO cells (Figures 7B,C). Together, these findings indicated that JAM-A promotes the recruitment of RhoGDI1 to cell-cell contacts in CHO cells.

To test if JAM-A recruits RhoGD1 through a direct interaction, we performed peptide pulldown assays with biotinylated peptides representing the cytoplasmic tail of JAM-A and *in vitro* translated RhoGDI1. Recombinant RhoGDI1 did not interact with JAM-A under these conditions (Supplementary Figure S5) suggesting that the recruitment of RhoGDI1 in cells is indirect and mediated through a JAM-A-associated cytoplasmic protein. Together, these findings indicated that the cell adhesion receptor JAM-A can promote the recruitment of RhoGDI1 to cell-cell contacts.

To test a potential cross-talk of RhoGDI1 and JAM-A we analyzed JAM-A expression levels in RhoGDI1-depleted cells by Western blot analysis. We observed no difference in the expression levels of JAM-A in RhoGDI1-depleted cells (Supplementary Figure S6A). We also analyzed the levels of RhoGDI1 in Eph4 cells ectopically expressing JAM-A, either JAM-A/WT or JAM-A mutants with mutations in residues involved in phosphorylation (JAM-A/S285A, JAM-A/Y281F) (Ozaki et al., 2000; Iden et al., 2012; Fan et al., 2019; Kummer et al., 2022). The levels of RhoGDI1 were unchanged in these cells (Supplementary Figure S6B). These findings suggested that JAM-A and RhoGDI1 do not regulate each other in a mutual manner.

## Discussion

The establishment of intercellular junctions and their maturation during cellular polarization requires the activity of Rho family small GTPases. The levels of active Rho GTPases must be precisely balanced as either too much or too little activity could disrupt junctional integrity. In this study, we have analyzed the role of RhoGDI1, which mainly binds members of the Rho and Rac subfamilies (Ahmad Mokhtar et al., 2021). We found that several distinct steps of junction formation in polarized epithelial cells are affected by depletion of RhoGDI1.

First, when cells were cultured on micropatterns (1D kinematic assays), we observed an increase in the fraction of cells that failed to stop migration when they encountered other cells but instead migrated across the other cell (Figure 4). We attribute this observation to an impaired inhibition of Rac1 activity upon contact formation. Rac1 activity is required at initial sites of cell-cell contacts to activate the PAR—aPKC complex but then is quickly downregulated at those sites, most likely to prevent a continuous protrusive activity to limit motility and prevent continuous migration (Ehrlich et al., 2002; Yamada and Nelson, 2007; Kitt and Nelson, 2011; Roycroft and Mayor, 2016). Rac1 inhibition at the leading edge of the migrating cells is an intrinsic component of type II CIL, defined as the cessation of movement in the direction of contact (Carter, 1967). Our observations, thus, suggest that RhoGDI1 contributes to the downregulation of Rac1 activity when cells collide to inhibit motility and prevent a continuous migratory activity.

Second, we observed a delay in the maturation of cell-cell junctions after CS-induced new junction formation (Figure 2A) as well as during the formation of a confluent cell monolayer from sparsely seeded cells (Figures 2B–D). This delayed junction maturation could be caused by the misregulation of several small GTPases in the absence of RhoGDI1. Active Rac1 is required during expansion of cell-cell junctions (Yamada and Nelson, 2007), and both active Rac1 and RhoA are required for the maturation of primordial junctions into fully matured junctions containing AJs and TJs (Terry et al., 2011; Reyes et al., 2014; Breznau et al., 2015; Priya et al., 2015). The delayed maturation of cell-cell contacts after RhoGDI1 depletion is, thus, most likely the consequence of imbalanced activities of both Rac1 and RhoA.

Third, RhoGDI1 depletion resulted in a defect in the formation of the epithelial barrier (Figure 3). This defect was not observed under steady state conditions but only when cells were subjected to a CS to induce new junction formation which suggested that RhoGDI1 is required to balance the activities of Rac1 and/or RhoA during the development of epithelial TJs but not at steady state. Under steady state conditions the levels of active RhoA and Rac1 are relatively stable (Priya et al., 2015; Priya et al., 2017), suggesting that RhoGDI1 activity is not required at steady state. Alternatively, the remaining low levels of RhoGDI1 in the RhoGDI1-depleted cells could be sufficient for the regulation of low dynamics of RhoA and Rac1 activities under steady state conditions. The possibility of RhoGDI1-independent mechanisms of RhoGTPase regulation also exists. For example, RhoGDI3 which is widely expressed and which has an interaction profile among Rac and Rho subfamily members that is very similar to RhoGDI1 (Ahmad Mokhtar et al., 2021) could compensate for the low RhoGDI1 levels at steady state. Finally, vesicle trafficking could account for RhoGTPase activity in RhoGDI1-depleted cells at steady state (Slaughter et al., 2009).

Fourth, we observed an altered migratory behavior of collectively migrating cells after depletion of RhoGDI1 (Figure 5A). During collective cell migration, leader cells positioned at the front of the migrating collective respond to chemical cues by increased protrusive activity at their leading edge and increased actomyosin-based contractility at their rear end (Scarpa and Mayor, 2016). Through cadherin-based intercellular junctions, the forces generated by the leader cells are transmitted to the follower cells (Bazellieres et al., 2015; Ladoux and Mege, 2017). At the same time, the follower cells generate protrusions in the direction of migration, so-called cryptic lamellipodia (Farooqui and Fenteany, 2005; Ozawa et al., 2020). The regulation and coordination of these different processes requires the activity of several Rho GTPases not only at cell-matrix adhesions but also at cell-cell adhesions (Zegers and Friedl, 2014). It is, thus, not surprising that depletion of RhoGDI1 results in an altered migration behavior of cell collectives. Of note, we observed an increased migration velocity after RhoGDI1 depletion which indicates that the regulation of Rho GTPases by RhoGDI1 is required to limit the speed of migration of a cellular collective.

One intriguing observation is that expression of the cell adhesion receptor JAM-A results in the localization of RhoGDI1 to cell-cell contacts in CHO cells (Figure 6; Figure 7). These observations suggest that JAM-A contributes to the junctional localization of RhoGDI1 in epithelial cells. Intriguingly, many steps of junction formation that are affected by RhoGDI1 depletion are also affected by JAM-A depletion, including CIL, junction maturation, and epithelial barrier development (Iden et al., 2012; Kummer et al., 2022). It will, therefore, be important to understand the molecular mechanism through which JAM-A contributes to the junctional localization of RhoGDI1. We did not observe an interaction of *in vitro* expressed RhoGDI1 with the cytoplasmic tail of JAM-A in peptide pulldown assays suggesting that the recruitment does not involve a direct interaction with JAM-A. We hypothesize that RhoGDI1 interacts with a scaffolding protein associated with JAM-A. Scaffolding proteins are characterized by several protein—protein interaction domains which can interact with integral membrane proteins, other scaffolding proteins and proteins involved in signaling (Liu and Fuentes, 2019; Rouaud et al., 2020). The interaction of RhoGDI1 with a scaffolding protein that can interact with JAM-A and possibly with additional cytoplasmic proteins localized at cell-cell contacts (Ebnet, 2017) would explain why RhoGDI1 is recruited by JAM-A in the absence of a direct interaction.

Several other cell-cell contact- or cell-matrix adhesions-localized receptors have been found to regulate the localization of RhoGDI1 at sites of cell-cell adhesion. For example, in human cancer cell lines ephrinB1 interacts with RhoGDI1 through its cytoplasmic domain which limits the activity of RhoA in the absence of ephrinB1 interaction with the EphB2 receptor (Cho et al., 2018). In keratinocytes, α2β1 integrin localized at cell-cell adhesions negatively regulates the localization of Tyr156-phosphorylated RhoGDI1 at cell-cell junctions (Howden et al., 2021). Since Tyr156-phosphorylation decreases the ability of RhoGDI1 to interact with Cdc42 (DerMardirossian et al., 2006), junction-associated α2β1 integrin prevents excessive Cdc42 activity, which is necessary for the stabilization of AJs (Howden et al., 2021). In astrocytes and glioblastoma cells RhoGDI1 interacts with the cytoplasmic domain of the β8 integrin subunit of the αvβ8 heterodimer, which limits the activity of Rac1 and Cdc42 (Reyes et al., 2013; Lee et al., 2015). It is likely that RhoGDI1 contributes to the regulation of local RhoGTPase activities in specific membrane microdomains, and it will thus be important to understand the molecular mechanisms that regulate the recruitment of RhoGDI1 to these specific subdomains.

## Materials and methods

### Cell culture and transfections

Eph4 cells (ATCC #CRL-3063, kindly provided by Dr. R. Windoffer, RWTH Aachen) and HEK293T cells (ATCC #CRL-3216) were cultivated in DMEM high glucose medium (SA #D5671) containing 10% FCS, 2 mM glutamine, 100 U/mL penicillin and 100 U/mL streptomycin. CHO^dhfr−^ cells (Urlaub and Chasin, 1980) were grown in αMEM medium (SA #M0450) containing 10% FCS, 2 mM glutamine, 100 U/mL penicillin and 100 U/mL streptomycin. CHO cells stably transfected with JAM-A were grown in medium supplemented with blasticidin (7 μg/mL, CHO-JAM-A) (Ebnet et al., 2001). Stable LifeAct-EGFP (LA-EGFP) or LifeAct-mCherry (LA-mCherry) expressing Eph4 cells were generated by lentiviral transduction and selection in growth media containing 100 μg/mL zeocin or 1 μg/mL puromycin, respectively, as previously described (Tholmann et al., 2022). All cell lines used in this study were routinely tested and found to be negative for *mycoplasma* contamination.

### Antibodies and reagents

The following antibodies were used: rat mAb anti ZO-1, R40.76 (SA #MABT11, immunofluorescence (IF) 1:500); mouse mAb anti ZO-1 (Invitrogen #33-9100, IF 1:500); rabbit pAb anti-ZO-2 (Invitrogen #71-1400, IF 1:500); mouse mAb anti RhoGDI1 (Santa Cruz Biotechnology #sc-373724, IF 1:500); rabbit pAb anti RhoGDI1 (Cell Signaling Technology (CST) #2564, Western blot (WB) 1:500, IF 1:500); rat mAb anti E-cadherin (SA #MABT26, IF 1:500); rat mAb anti JAM-A (H2O2-106-7-4, (Malergue et al., 1998), IF: 1:500); rabbit pAbs anti mJAM-A (Affi1165, IF 1:500; Affi798 WB 1:700); mouse mAb anti Cdc42 (BD Biosciences (BD) #610928, WB 1:250); mouse mAb anti Rac1 (Millipore #05-389, WB 1:500); mouse mAb anti RhoA (BD #610991, WB 1:250); rabbit pAb anti Vimentin (proteintech #10366-1-AP, WB 1:1000); rabbit pAb anti Slug (mouse mAb, SantaCruz #sc-166476, WB 1:500); rabbit pAb anti GAPDH (Thermo Fisher Scientific #PA1-987, WB 1:3000); mouse mAb anti α-Tubulin (SA #T5168, WB 1:10.000); rabbit pAb anti-Flag (SA, F7425, WB 1:1000). The following reagents were used: 2,4,diamidino-2-phenylindole (DAPI, SA #D9542); Fibronectin (FN, SA #F2006); FITC-dextran 4 kDa (SA #FD4); FITC-dextran 70 kDa (SA #FD70S); Phalloidin-iFluor 594 (Abcam, #ab176757; dil 1:1000).

### RNA interference, plasmid vectors and constructs

The following shRNAs were used: mRhoGDI1 shRNA (5′-GCC​TGG​CCT​GTC​AGT​ATT​TAT-3′) in pLKO.1 (Horizon Discovery, Cambridge, United Kingdom, #RMM3981-201831009; clone ID TRCN0000106160) (Gupta et al., 2013). Scrambled shRNA (5′- CCT​AAG​GTT​AAG​TCG​CCC​TCG-3′) in pLKO.1 was a gift from David Sabatini (Addgene plasmid # 1864; http://n2t.net/addgene1864; RRID:Addgene_1864) (Sarbassov et al., 2005; Kummer et al., 2022). RhoGDI1 constructs: Myc-hRhoGDI1 full length (AA 1–204) in pCDH-CMV-MCS-EF1-Hygro (System Bioscience, Mountain View, CA, United States); EGFP-hRhoGDI1 full length (AA 1–204) in pEGFP-C1. Csk constructs: Flag-Csk/SH2 (AA 50-210) in pcDNA3 (Kummer et al., 2022). ZO-1 constructs: EGFP-ZO-1 (kindly provided by Dr. Junichi Ikenouchi) contains the ZO-1 coding sequence with an N-terminal fusion of EGFP. ZO-2 constructs: pEGFP-C3-ZO-2 (a gift from Marius Sudol, Addgene plasmid #27422; http://n2t.net/addgene:27422; RRID:Addgene_27422) contains the ZO-2 coding sequence with an N-terminal fusion of EGFP (Oka et al., 2010). JAM-A constructs in pCDH-CMV-MCS-EF1-Puro (System Bioscience, Mountain View, CA, United States): Flag-JAM-A/WT (AA 26-300), Flag-JAM-A/S285A (AA 26-300, Ser_285_Ala), Flag-JAM-A/Y281F (AA 26-300, Tyr_281_Phe). LA-EGFP in lentiviral vector pFUGW was provided by Dr. H. Schnittler, LA-mCherry in lentiviral vector pLV-PGK-Puro was provided by Dr. H. Farin.

### *In vitro* binding experiments and Western blot analysis

*In vitro* binding experiments were performed with biotinylated peptides immobilized on streptavidin beads (Sigma-Aldrich) as previously described (Kummer et al., 2022). For *in vitro* interactions the putative partner proteins (prey) were translated *in vitro* using the TNT T7-coupled reticulocyte lysate system (Promega Corp., Madison, WI) as described by the manufacturer. The translation reactions were incubated with 0.5 µg biotinylated peptide immobilized on streptavidin beads for 2 h at 4°C under constant agitation in buffer B (10 mM Hepes-NaOH (pH7.4), 100 mM KCl, 1 mM MgCl_2_, 0.1% Triton X-100). After five washing steps in buffer B bound proteins were eluted by boiling for 5 min in SDS sample buffer, subjected to SDS-PAGE and analyzed by Western blot analysis. For the generation of cell lysates, cells were washed 2x with PBS^−/−^ on ice, harvested in 2x Laemmli sample buffer (100 µL per 1 × 10^6^ cells (Laemmli, 1970), using a cell scraper, and boiled for 10 min at 95°C). Alternatively, cells were harvested in NP40-lysis buffer (50 mM Tris-HCl, 150 mM NaCl, 1% NP40, pH = 7.5) using a cell scraper, followed by overhead rotation for 1 h at 4°C. After centrifugation (14.000 rpm, 4°C, 15 min) the supernatants were diluted 1:3 with 3x sample buffer and boiled for 10 min at 95°C. Cell lysates were subjected to SDS-PAGE and analyzed by Western blotting with near-infrared fluorescence detection (Odyssey Infrared Imaging System Application Software Version 3.0 and IRDye 800CW-conjugated antibodies; LI-COR Biosciences, Bad Homburg, Germany). Peptide pulldown and Western blot experiments shown in the figures are representative of at least three independent experiments.

### Ca^2+^-switch (CS) experiments and analysis of junction formation

For Ca^2+^-switch (CS) experiments, confluent Eph4 cells were incubated in PBS, 5 mM EDTA (PBS-EDTA) for 3 × 5 min, as previously described (Beeman et al., 2009). To induce new cell-cell contact formation, the PBS-EDTA solution was replaced by regular culture medium. The rate of junction assembly was determined by measuring the accumulation of ZO-1 at cell-cell contacts using ImageJ software (http://rsb.info.nih.gov/ij/disclaimer.html). Briefly, a binary image of the ZO-1 signal was generated and the area of ZO-1 signal was computed using a Macro (provided as Appendix to this article). The computed area was normalized to the mean ZO-1 signal intensity observed in cells grown under normal Ca^2+^ conditions. For each condition, at least ten fields of view containing an average number of 63 cells were analyzed per experiment. Each experiment was performed at least three times. Mean values and standard deviations were calculated from three independent experiments.

### Immunofluorescence microscopy

For immunofluorescence microscopy, cells were grown on FN-coated (5 μg/mL) glass slides and fixed with 4% paraformaldehyde (PFA, SA) for 10 min at room temperature (RT). Cells were permeabilized by incubation with PBS, 0.5% Triton X-100 for 10 min at RT, followed by three washes with PBS, 100 mM glycine. Unspecific binding sites were blocked by incubation for 1 h in blocking buffer (10% FCS, 0.2% Triton X-100, 0.05% Tween-20, 0.02% BSA in PBS). Primary antibodies were applied overnight at 4°C in blocking buffer. After washing three times with PBS, the cells were incubated with fluorochrome (AlexaFluor488, AlexaFluor594 or AlexaFluor647)-conjugated, highly cross-adsorbed secondary antibodies (Invitrogen) and DAPI for 2 h at RT. The samples were washed thoroughly with PBS and mounted in fluorescence mounting medium (Mowiol 4-88, SA #81381). Immunofluorescence microscopy was performed using the confocal LSM800 microscope (Carl Zeiss, Jena, Germany) equipped with a Plan-Apochromat x 63/1.4 oil differential interference contrast objective (Carl Zeiss). Image processing and quantification was performed using ZEN 2012 (Carl Zeiss), ImageJ (National Institutes of Health, Bethesda, MD), and Imaris (Bitplane, Version 9.1.2) softwares.

### Transepithelial electrical resistance

The transepithelial electrical resistance (TER) of Eph4 cells was analyzed essentially as described previously (Iden et al., 2012). Briefly, cells were grown on FN-coated polycarbonate filters (0.4 μm pore size, Corning #3413) in 24-well tissue culture dishes. After reaching confluence, cells were subjected to a CS. TER was monitored online over a period of 40 h using an automated multi-well cellZscope^®^ device (nanoAnalytics, Münster, Germany). This device was placed within the CO_2_-incubator, and online recordings of the TER were taken in 15-min intervals resulting in a smooth curve when plotted *versus* time over a period of 40 h. For statistical analysis the TER-values were normalized to the corresponding values before depleting the cells of calcium. For each clone and each experiment at least three filters were analyzed. Each experiment was performed four times. Mean values and standard deviations were calculated from four independent experiments.

### Paracellular diffusion

For the analysis of FITC-dextran permeability cells were seeded on FN-coated polycarbonate filters (0.4 μm pore size, Corning #3413). After reaching confluence, cells were subjected to a CS as described above. The permeability for FITC-dextran was analyzed by adding 4 kDa FITC dextran (2.5 mg/mL) or 70 kDa FITC dextran (2.5 mg/mL) to the upper compartment of the filters. After 2 h, the fluorescence in the lower compartment was analyzed using a fluorescence reader (emission at 520 nm, CLARIOstar^®^, BMG Labtech). For each clone and each experiment at least three filters were analyzed. Each experiment was performed at least three times. Mean values and standard deviations were calculated from three independent experiments.

### 1D micropattern assay (1D kinematic assay)

For one-dimensional (1D) collision assays (1D kinematic assays) (Scarpa et al., 2013; Schwietzer et al., 2023), chips with micropatterns of linear tracks of 5 µm width (CYTOOchips™ Motility Ax18, CYTOO INC, Grenoble, France) were used. Co-cultures of either RhoGDI1 KD Eph4 (expressing RhoGDI1 shRNAs) or control Eph4 cells (expressing scrambled shRNAs) with WT Eph4 cells were seeded on FN-coated micropatterned stripes at 5 × 10^4^ cells/mL. Cells were allowed to adhere to the surface for 2 h, then observed by live cell microscopy over a period of 15 h with image acquisition at 10-min intervals. Live cell microscopy was performed using the LSM780 (Carl Zeiss) confocal microscope equipped with a Plan-Neofluar × 20/0.5 objective at 37°C in normal culture medium. Post-collision cell behavior was categorized as follows: Type −2 (opposite migration), type −1 (anergy, i.e., stop of migration without cell-cell contact formation), type 0 (cell-cell contact formation), type +1 (continuous migration, i.e., migration across collided cell). Statistical analyses were performed with data from N = 4 independent experiments using Chi-Square test and *post hoc* Bonferroni corrections. Single cell migration assays were performed as described for 1D collision assays. Migration velocity and track displacement of individual cells were analyzed semi-automatically using the TrackMate Plugin for ImageJ. Migration directionality was calculated by dividing the displacement (Euclidian distance) at the end of the observation period through the entire migrated track. Statistical analyses were performed with data from N = 3 independent experiments using a two-sided Mann-Whitney U-test.

### Collective cell migration

For the analysis of collective cell migration of epithelial cells, a monolayer expansion assay was used in which collective cell migration is triggered by a free surface (Poujade et al., 2007; Das et al., 2015). Eph4 cells were seeded in different compartments of FN-coated microscope slides (Ibidi µ-Slide two well glass bottom, Ibidi #80287) separated by a removable silicone stamp (Ibidi Culture-Inserts 2 Well for self-insertion, Ibidi #80209). Cells were grown for 72 h to confluency before removal of the stamp to trigger sheet migration. Pictures were taken directly after removal of the stamp and 8 h later by using an EVOS digital inverted microscope. The collective cell migration speed was calculated as the mean distance between the initial position of the cell sheet’s front and its position at the end of the observation time using ImageJ. Briefly, the cell-free area measured at the end of the observation period (t_1_ = 8h) was subtracted from the cell-free area at the beginning (t_0_) resulting in the total area covered by migrated cells. This area was divided by the height of the gap resulting in the total distance that the cells had migrated. To take into account that the cells close the gap from both sides the total distance was divided by two resulting in the distance migrated by a single sheet. The migration speed of the cellular collective was calculated by dividing the distance of a single sheet by the observation time and is given in µm/min. Experiments were performed at least three times with four separate migration chambers (biological replicates) per experiment.

### Cell junction analysis

Cell junction morphology analysis was done in Eph4 cells stained for ZO-1. In analogy to previous studies in endothelial and epithelial cells (Taguchi et al., 2011; Vion et al., 2020), cell-cell junctions were classified on the basis of their morphology as strongly serrated (more than 50% of ZO-1 fluorescence signal oriented perpendicular to the cell junction), weakly serrated (less than 50% of ZO-1 fluorescence signal oriented perpendicular to the cell junction) and linear (straight and thick ZO-1 fluorescence signal). At least 10 randomly chosen fields of view per condition were chosen for analysis, and at least 187 cell-cell junctions per condition were analyzed by blinded visual inspection. Analysis of EGFP-RhoGDI1, EGFP-ZO-1 and EGFP-ZO-2 recruitment by JAM-A in CHO cells (Figure 7) was performed by visual identification of cell-cell contact sites. EGFP-RhoGDI1-, EGFP-ZO-1- and EGFP-ZO-2-transfected cells were identified on the basis of the cytoplasmic EGFP fluorescence signal. Only cell pairs in which both cells were in clear contact without fluorescence-free gaps were counted. JAM-A-based cell-cell contacts were counted positive for the transfected EGFP fusion proteins when the EGFP fluorescence signals co-localized with the JAM-A-based fluorescence along the cell-cell contact sites. Data is represented as fraction of cell-cell contacts positive for the EGFP fusion protein. At least 115 cell-cell junctions per condition were analyzed by blinded visual inspection. For the analysis of F-actin localization, Eph4 cells were grown on 6.5 mm Transwell^®^ with 0.4 µm pore polycarbonate membrane inserts (Corning #3413) for 3 days and stained for ZO-1 and F-actin. Cells were analyzed by confocal microscopy with approx. 30 confocal sections taken per field of view. In each experiment at least 10 randomly selected fields of view each containing approximately 100 cells were used for analysis. Fluorescence intensities for ZO-1 and F-actin were analyzed using Imaris software. Data shows the fraction of F-actin fluorescence colocalized with ZO-1 fluoresecence (indicated by Pearsons’s correlation coefficient) (Dunn et al., 2011). Data is derived from three independent experiments.

### Statistics

Results are expressed as arithmetic means ± SD as indicated in the figure legends. To test the normality of data, D’Agostino-Pearson normality test was used. Data were statistically compared using unpaired, two-tailed Student’s t-test. For data with categorical variables Fisher’s test (two possible outcomes) or Chi-Square test with *post hoc* Bonferroni corrections (more than two possible outcomes) were used. Statistical analyses were performed using GraphPad Prism version 6 (GraphPad Software, San Diego, CA). *p*-values are indicated as follows: *p < 0.05, **p < 0.01, ***p < 0.001 and ****p < 0.0001.

## Data Availability

All relevant data is contained within the article. The original contributions presented in the study are included in the supplementary material. Additional data supporting the findings of this article are available from the corresponding author upon reasonable request.

## Abbreviations

AA, amino acid; CS, Ca2+ switch; IF, immunofluorescence; JAM-A, junctional adhesion molecule-A; RhoGDI1, Rho GDP-dissociation inhibitor 1; JAM-A, junctional adhesion molecule-A; RhoGDI1, Rho GDP dissociation inhibitor 1; TER, transepithelial electrical resistance.

## Appendix

ImageJ Macro for the analysis of junction formation.

//settings
run (“Options…”, “iterations = 1 count = 1 black”);
setOption (“BlackBackground”, true);
run (“Set Measurements…”, “area limit display redirect = None decimal = 3”);
//split channels
title = getTitle ();
run (“Split Channels”);
//count nuclei selectWindow (“C3-”+ title);
run (“Median…", “radius = 3”);
run (“Auto Threshold”, “method = Huang white”);
run (“EDM Binary Operations”, “iterations = 4 operation = close”);
run (“Adjustable Watershed”, “tolerance = 0.3”);
run (“Analyze Particles…”, “size = 4-Infinity show = Outlines display summarize add”);
//measure area of contacts
selectWindow (“C1-”+ title);
run (“Median…”, “radius = 1 stack”);
setAutoThreshold (“Default”); run (“Threshold…");
//setThreshold (80, 255);
setThreshold (80, 255);
waitForUser (“Please adjust Threshold");
setOption (“BlackBackground”, true); run (“Convert to Mask”, “method = Default background = Light black”);
waitForUser (“delete particles”);
setOption (“BlackBackground”, true);
run (“Convert to Mask”, “method = Default background = Light black”);
run (“Analyze Particles…”, “size = 0.00-infinity circularity = 0.00–0.4 show = Masks display summarize add”);
run (“Measure”);
