# Supplementary material for: RhoGDI1 regulates cell-cell junctions in polarized epithelial cells
Source: Front Cell Dev Biol. 2024 Jul 17;12:1279723. doi: 10.3389/fcell.2024.1279723 (PMC11288927; doi:10.3389/fcell.2024.1279723)

Source data for Fig. S1

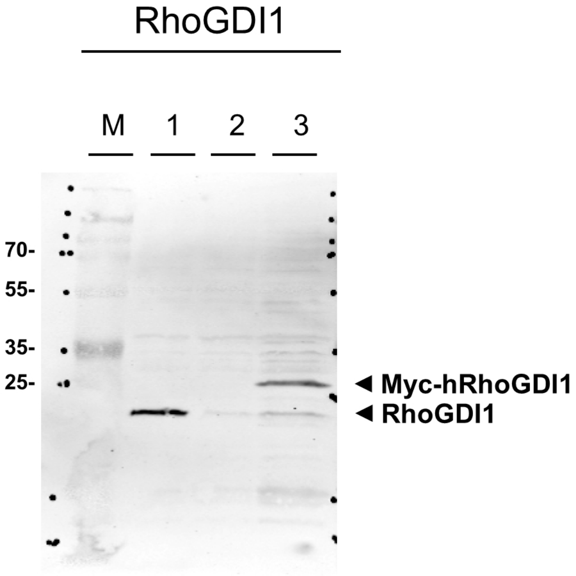

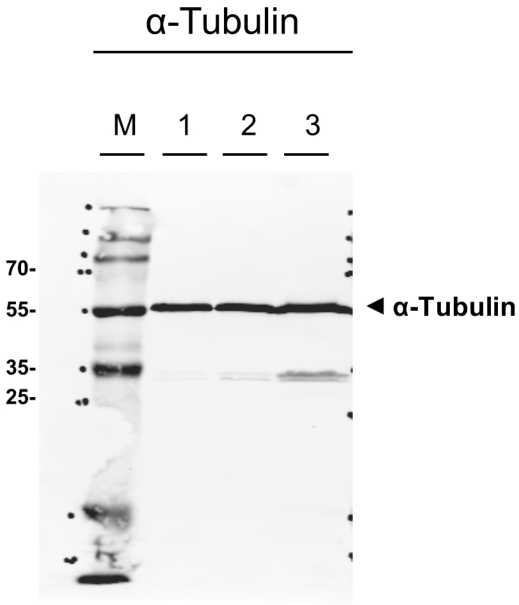

1: Scr shRNA
2: RhoGDI1 shRNA
3: RhoGDI1 shRNA + Myc-hRhoGDI1
M: Marker

Source data for Fig. S2

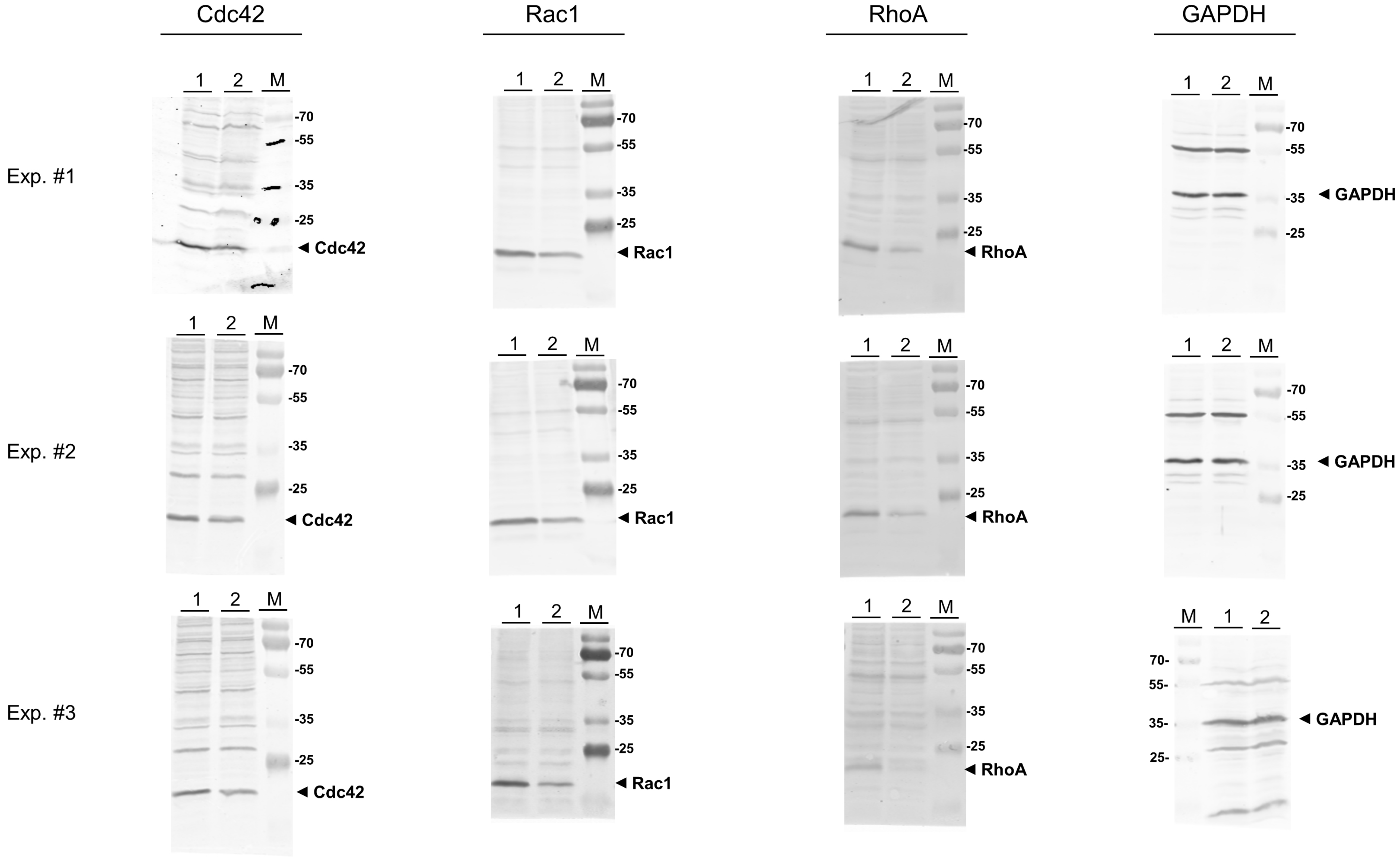

1: Scr shRNA
2: RhoGDI1 shRNA
M: MW Marker

Source data for Fig. S3

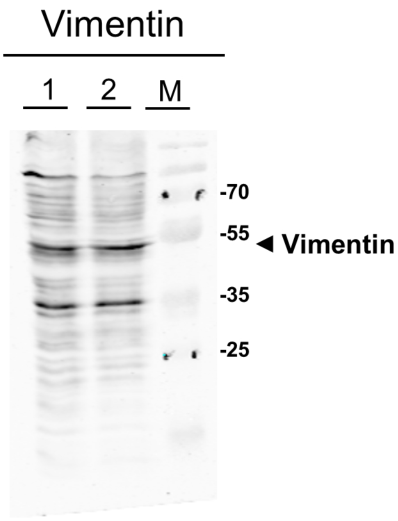

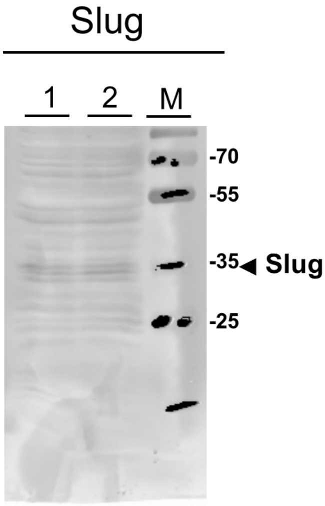

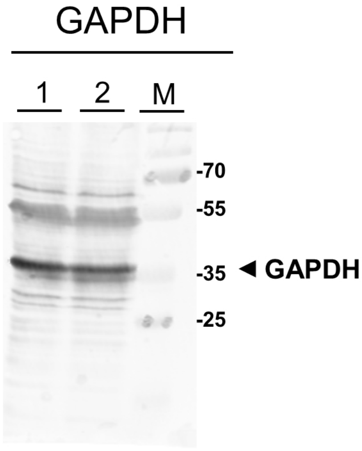

1: Scr shRNA
2: RhoGDI1 shRNA
M: MW Marker

Source data for Fig. S5

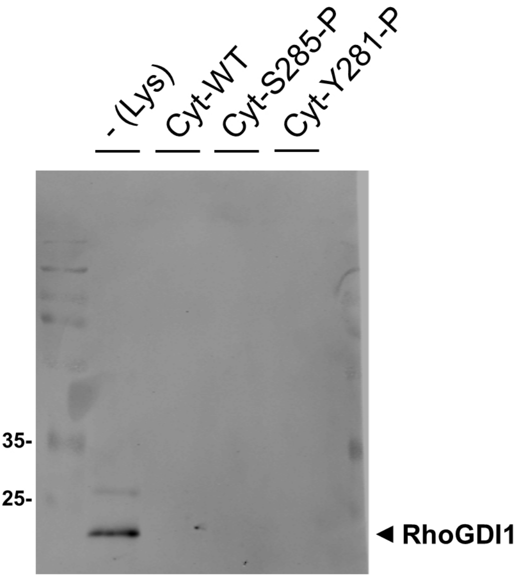

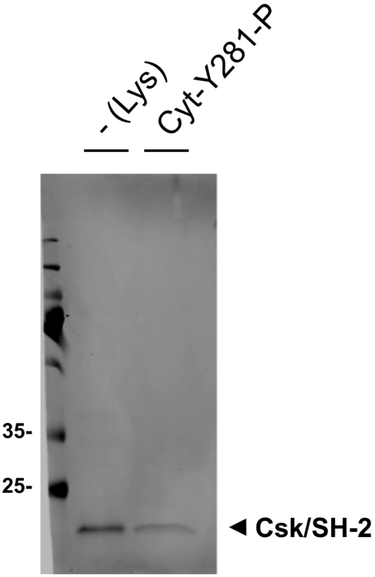

Source data for Fig. S6

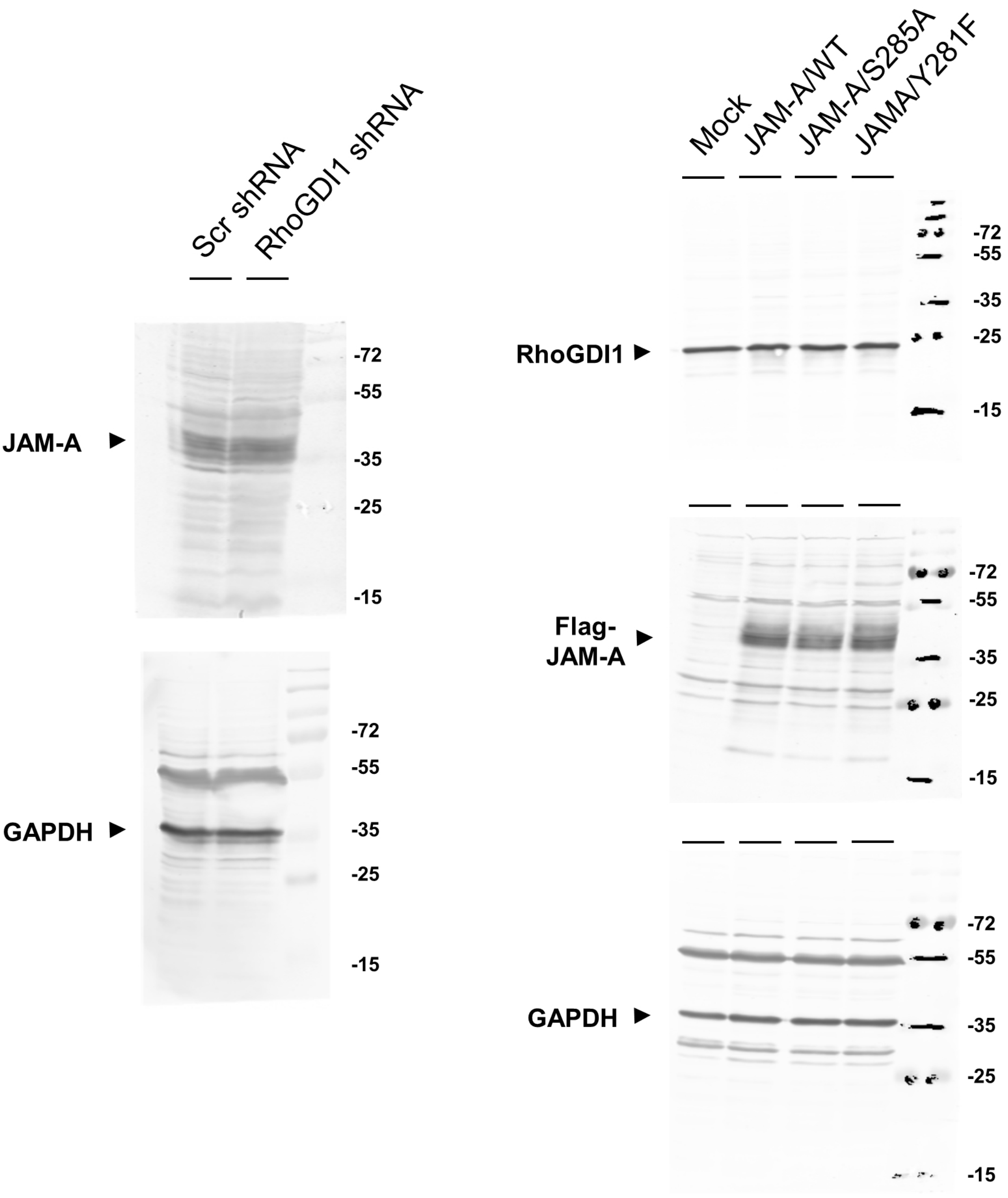

Supplement: Supplementary file 1 [file DataSheet2.PDF]
