# Supplementary material for: RhoGDI1 regulates cell-cell junctions in polarized epithelial cells
Source: Front Cell Dev Biol. 2024 Jul 17;12:1279723. doi: 10.3389/fcell.2024.1279723 (PMC11288927; doi:10.3389/fcell.2024.1279723)
Supplement: Supplementary file 2 [file DataSheet1.pdf]

Supplementary Material

RhoGDI1 regulates cell-cell junctions in polarized epithelial cells

Nicolina Wibbe[1], Tim Steinbacher[1], Frederik Tellkamp[2], Niklas Beckmann[1], Frauke Brinkmann[3], Manuel Stecher[3], Volker Gerke[3,4], Carien M. Niessen[5], Klaus Ebnet[1,4,*]

[1]Institute-associated Research Group "Cell adhesion and cell polarity", Institute of Medical Biochemistry, ZMBE; University Münster, Münster, Germany;
[2]Department Cell Biology of the Skin, University Hospital of Cologne, University of Cologne, Cologne, Germany; Cologne Excellence Cluster on Stress Responses in Aging-associated Diseases (CECAD), University of Cologne, Cologne, Germany; Present address: Institute for Genetics, University of Cologne, Cologne, Germany;
[3]Institute of Medical Biochemistry, ZMBE; University Münster, Münster, Germany;
[4]Cells-in-Motion Cluster of Excellence (EXC 1003 - CiM), University of Münster, Münster, Germany;
[5]Department Cell Biology of the Skin, University Hospital of Cologne, University of Cologne, Cologne, Germany; Cologne Excellence Cluster on Stress Responses in Aging-associated Diseases (CECAD), University Hospital of Cologne, University of Cologne, Cologne, Germany; Center for Molecular Medicine Cologne (CMMC), University Hospital of Cologne, University of Cologne, Cologne, Germany;

[*]Correspondence
Klaus Ebnet, PhD, Institute-associated Research Group "Cell adhesion and cell polarity", Institute of Medical Biochemistry, ZMBE, University of Münster, Von-Esmarch-Str. 56, D-48149 Münster, Germany
Tel.: +49-(0)251-8352127
FAX: +49-(0)251-8356748
e-mail: ebnetk@uni-muenster.de

Running title: RhoGDI1 and epithelial cell junctions

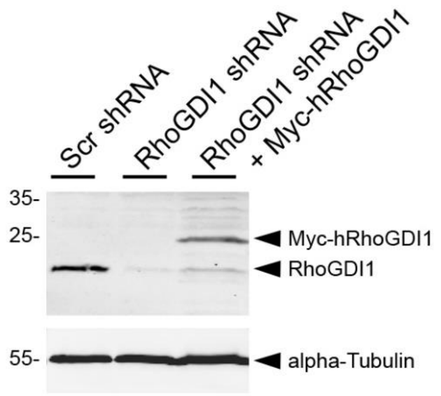

Figure S1: Western blot analysis of RhoGDI1 in Eph4 cells stably expressing a scrambled shRNA (Scr shRNA), a RhoGDI1 shRNA (RhoGDI1 shRNA), or a RhoGDI1 shRNA and a shRNA-insensitive Myc-tagged human RhoGDI1 construct (RhoGDI1 shRNA + Myc-hRhoGDI1). Western blot analysis for α-tubulin was used as loading control. Relative molecular weight (M_R) markers (in kDa) are indicated on the left.

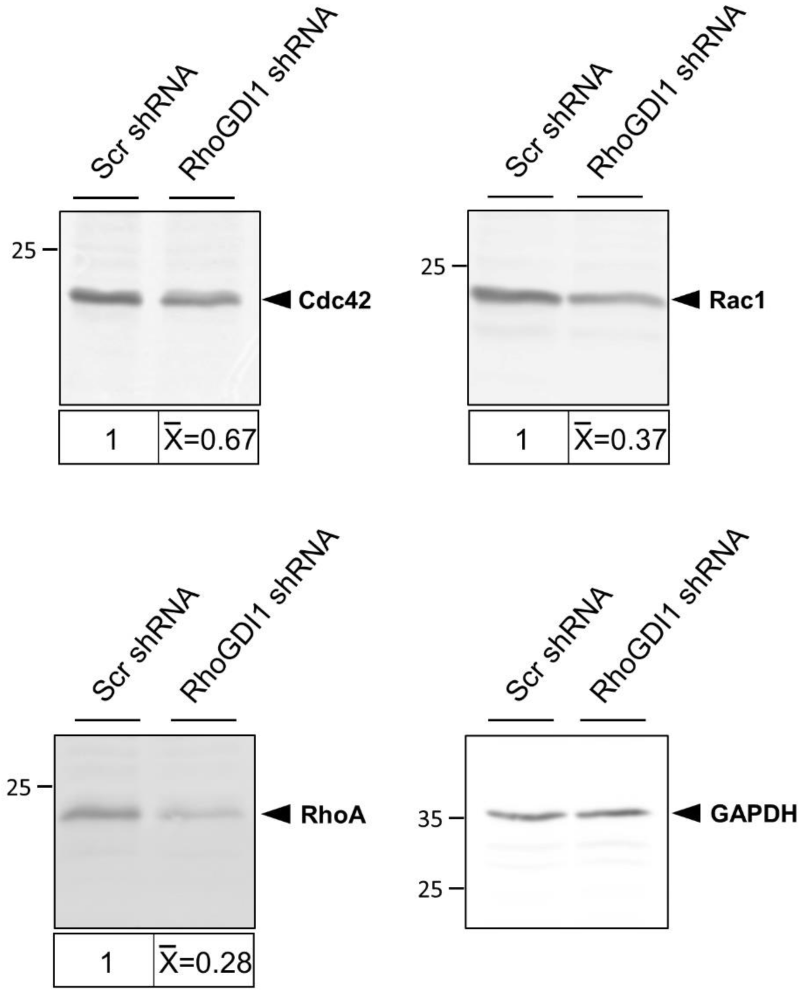

Figure S2: (**A**) Western blot analysis of Cdc42, Rac1 and RhoA in RhoGDI1-depleted Eph4 cells. Eph4 cells stably expressing a scrambled shRNA (Scr shRNA) or a RhoGDI1 shRNA (RhoGDI1 shRNA) were analyzed for the expression of Cdc42, Rac1 and RhoA as indicated. Western blot analysis for GAPDH was used as loading control. Relative molecular weight (M_R) markers (in kDa) are indicated on the left. Western blot signals were quantified using the Odyssey imaging system (LI-COR). Signal intensities were corrected for differences in GAPDH expression levels. Numbers below immunoblots indicate relative expression levels of the RhoGTPases in RhoGDI1-depleted cells vs control cells. Signals obtained for control samples were arbitrarily set to 1. Data represent arithmetic means of three independent experiments. Uncropped images of Western blots of the three experiments are displayed in the source file.

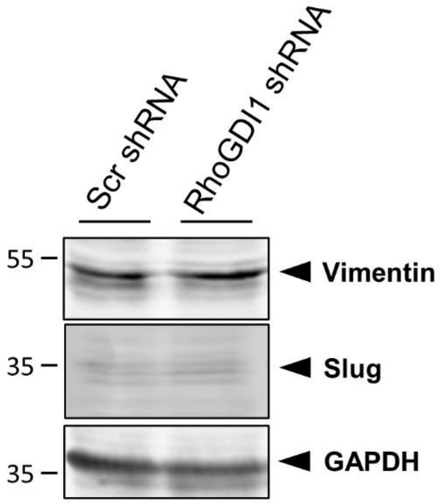

Figure S3: Western blot analysis of vimentin and Slug in RhoGDI1-depleted Eph4 cells. Eph4 cells stably expressing a scrambled shRNA (Scr shRNA) or a RhoGDI1 shRNA (RhoGDI1 shRNA) were analyzed for the expression of vimentin and Slug as indicated. Western blot analysis for GAPDH was used as loading control. Relative molecular weight (M_R) markers (in kDa) are indicated on the left.

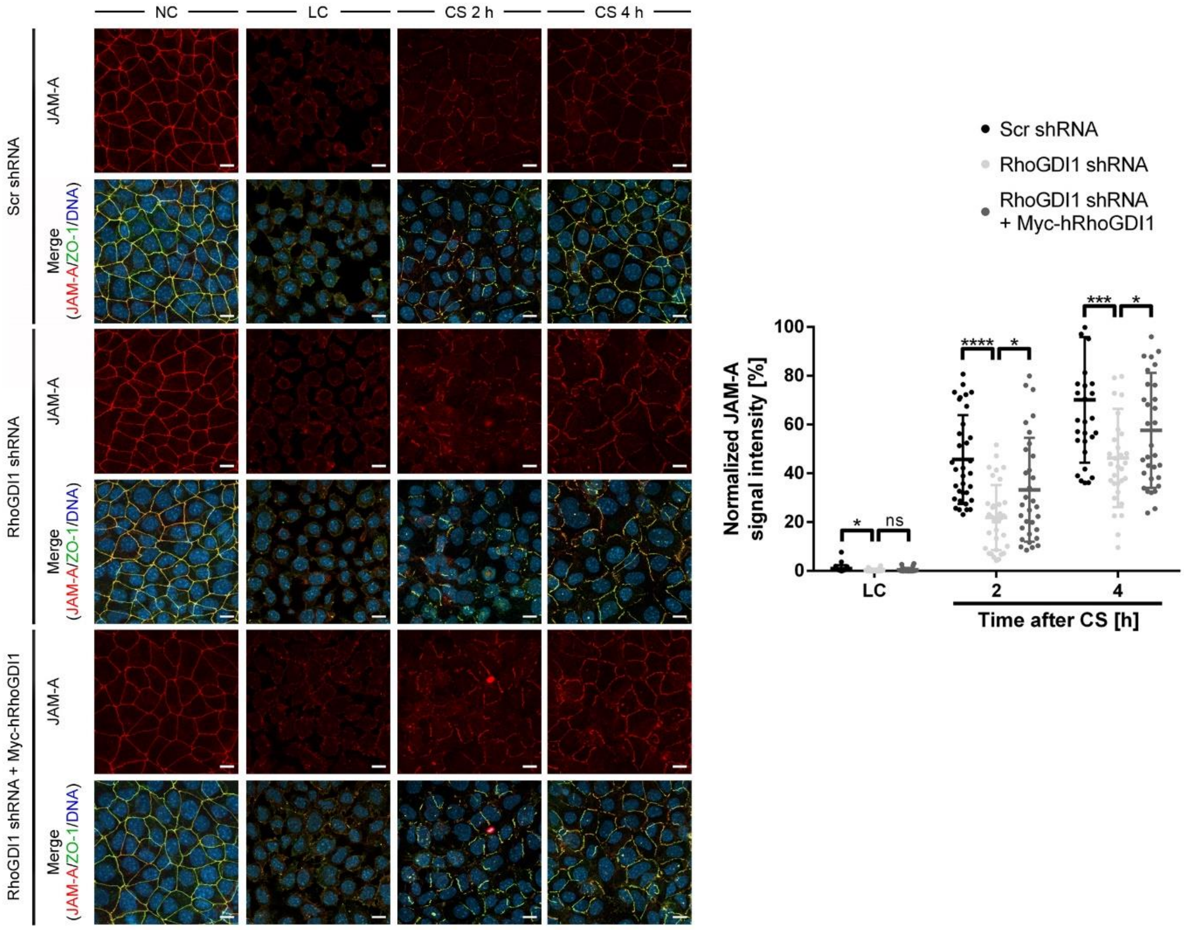

Figure S4: JAM-A recruitment during junction formation after depletion of RhoGDI1. Eph4 cells (Scr shRNA, RhoGDI1 shRNA, RhoGDI1 shRNA + Myc-hRhoGDI1) were cultured under normal Ca^{2+} conditions (NC) or under low Ca^{2+} conditions (LC) and either fixed immediately (LC) or after growth in normal medium (Ca^{2+} switch, CS) for 2 h or 4 h. Cells were stained for JAM-A (red fluorescence) and ZO-1 (green fluorescence; ZO-1 staining is shown in Fig. 5A). Merge pictures show overlays of red (JAM-A), green (ZO-1) and blue (DNA) fluorescence signals. Panels show representative immunofluorescence images of cells cultured under the indicated conditions. Scale bars: 10 µm. Right panel: Quantification of JAM-A localization at different time points after CS as indicated. The JAM-A IF intensities were analyzed using Image J software, data were normalized to the JAM-A intensities observed in cells grown under normal Ca^{2+} conditions (NC, 100%). Statistical analysis was performed with unpaired, two-tailed Student's t-test. Data is derived from at least 30 independent fields of view derived from three independent experiments. Data are presented as mean values ± SD. ns, not significant; *P<0.05, ***P<0.001, ****P<0.0001. Abbreviations: NC, normal Ca^{2+} conditions; LC, low Ca^{2+} conditions; CS, Ca^{2+} switch.

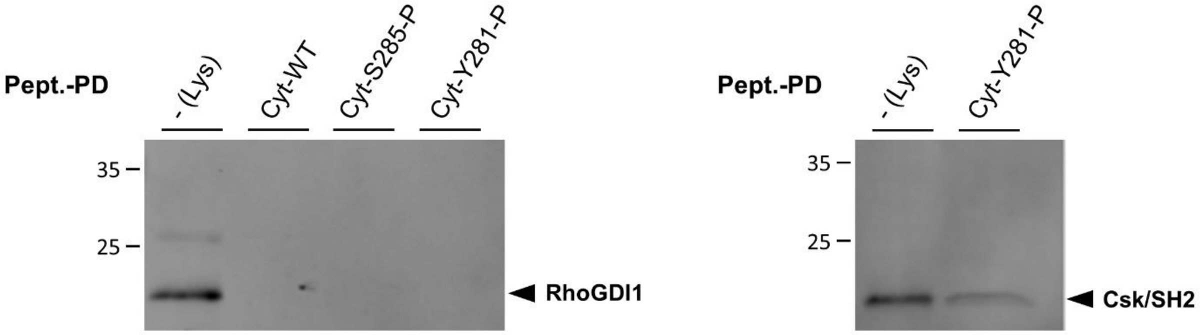

Figure S5: Biotinylated JAM-A cytoplasmic domain peptides, either the wildtype cytoplasmic domain (Cyt-WT) or the cytoplasmic domain phosphorylated at Ser285 (Cyt-S285-P) or at Tyr281 (Cyt-Y281-P) were incubated with in vitro translated recombinant Myc-RhoGDI1 (left panel) or Flag-Csk-SH2 (right panel, positive control). Bound proteins were analyzed by Western blotting. Abbreviations: Cyt, cytoplasmic; Lys, lysate; Pept.-PD, peptide pulldown.

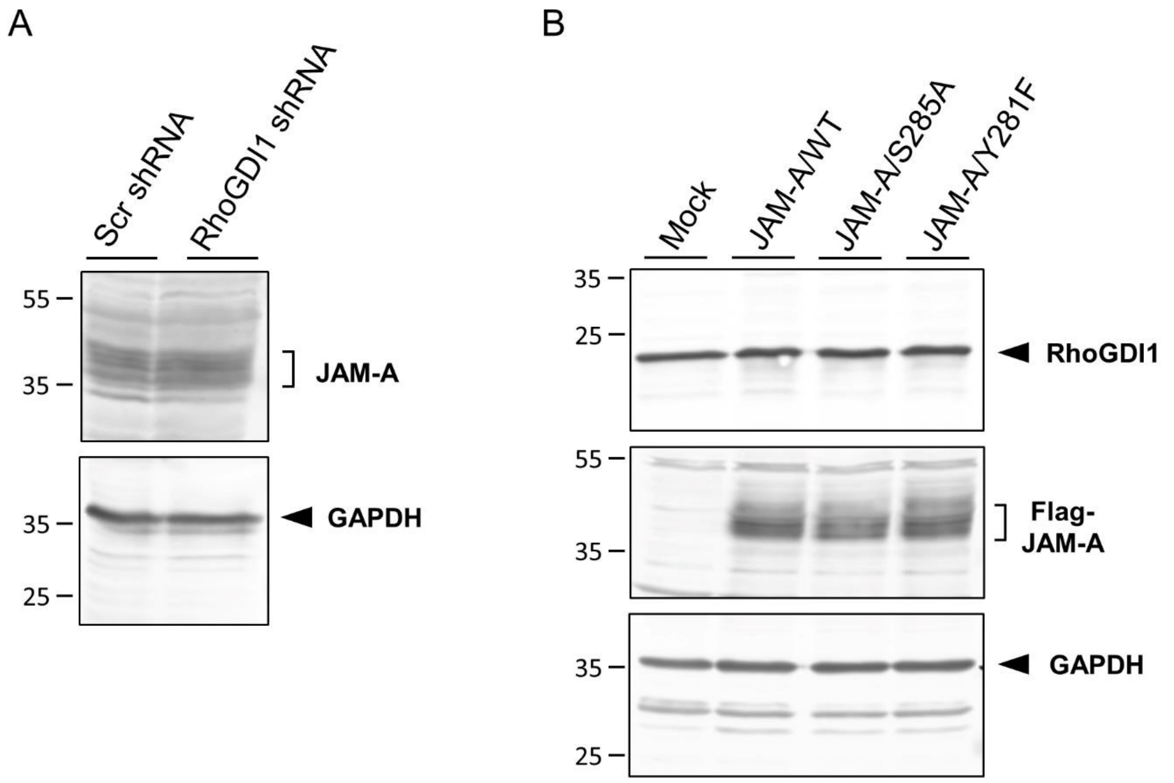

Figure S6: (**A**) Western blot analysis of JAM-A in RhoGDI1-depleted Eph4 cells. Eph4 cells stably expressing a scrambled shRNA (Scr shRNA) or a RhoGDI1 shRNA (RhoGDI1 shRNA) were analyzed for the expression of JAM-A as indicated. (**B**) Western blot analysis of RhoGDI1 in Eph4 cells expressing JAM-A constructs. Eph4 cells, transfected with either empty vector (Eph4_Mock) or with Flag-tagged JAM-A constructs including wildtype JAM-A (Eph4_JAM-A/WT), JAM-A with a Ser-to-Ala mutation at position 285 (JAM-A/S285A) or JAM-A with a Tyr-to-Phe mutation at position 281 (JAM-A/Y280F) were analyzed for the expression of RhoGDI1 or JAM-A constructs as indicated. Western blot analyses for GAPDH were used as loading controls. Relative molecular weight (M_R) markers (in kDa) are indicated on the left of each panel. Note that JAM-A runs as multiple bands (indicated by brackets).
